# The *cis*-regulatory logic integrating spatial and temporal patterning in the vertebrate neural tube

**DOI:** 10.1016/j.devcel.2025.06.029

**Published:** 2025-07-16

**Authors:** Isabel Zhang, Giulia L.M. Boezio, Jake Cornwall-Scoones, Thomas Frith, Elizabeth Finnie, Junyi Luo, Ming Jiang, Michael Howell, Robin Lovell-Badge, Andreas Sagner, James Briscoe, M. Joaquina Delás

**Affiliations:** 1 The Francis Crick Institute, London NW1 1AT, UK; 2 Laboratory for Molecular Cell Biology, University College London, Gower Street, London WC1E 6BT, UK; 3 Institute of Biochemistry, Friedrich-Alexander-Universität Erlangen-Nürnberg, Erlangen, Germany

## Abstract

The vertebrate neural tube generates a large diversity of molecularly and functionally distinct neurons and glia from a small progenitor pool. While the role of spatial patterning in organizing cell fate specification has been extensively studied, temporal patterning, which controls the timing of cell type generation, is equally important. Here, we define a global temporal program operating in progenitors throughout the mouse nervous systems that governs cell fate choices by controlling chromatin accessibility. Perturbation of this *cis*-regulatory program affects sequential cell fate transitions in neural progenitors and the identity of their progeny. The temporal program operates in parallel to spatial patterning, ensuring the timely availability of regulatory elements for spatial determinants to direct cell-type-specific gene expression. These findings identify a chronotopic spatiotemporal integration strategy in which a global temporal chromatin program determines the output of a spatial gene regulatory network resulting in the ordered allocation of cell type identity.

## Graphical abstract

**Graphical abstract:**
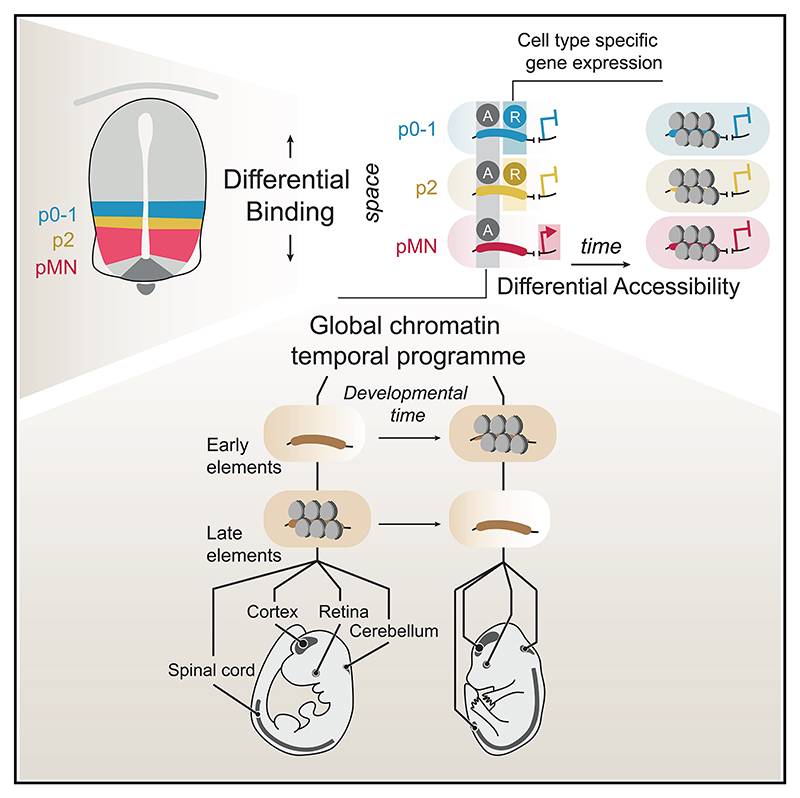

## Introduction

The developing vertebrate neural tube generates a remarkable diversity of neuronal and glial subtypes. This diversity is essential for neural circuit formation and function, yet how it arises during development remains incompletely understood. A gene regulatory network establishes spatial patterns of transcription factor (TF) expression in neural progenitors (NPs) that governs the identity and pattern of neuronal and glial subtype generation.^1–4^ However, spatial patterning alone fails to explain the full diversity of cell types. Individual progenitor domains produce molecularly distinct neurons and glia in a specific sequential order, markedly expanding neural cell diversity.^5–13^

The intersection of spatial and temporal identity not only increases molecular diversity but also organizes the arrangement of functionally distinct cell types.^14–18^ For example, long-range projection neurons in the spinal cord are generated prior to locally connected interneurons but the subtype of projection or local interneuron is determined by the position of generation.^19,20^ This suggests that spatial and temporal information are integrated within NPs to allocate the spatially and temporally appropriate neuronal subtype. However, how temporal patterning is executed in progenitors and how spatial and temporal information are integrated to direct cell fate decisions is unclear.

In the *Drosophila* nervous system, a set of spatial patterning TFs (STFs) establish chromatin accessibility configurations in different neuroblasts.^21^ These provide the context for cascades of temporal TFs (TTFs) to bind chromatin and specify different cell fates.^21–23^ This suggests a spatiotopic mechanism for the integration of spatial and temporal information: STFs determine the set of *cis*-regulatory elements (CREs) accessible in a particular neuroblast thereby governing the binding profile of TTFs and hence the specific gene expression program activated.

In the vertebrate nervous system, the transition from the production of early-born neurons to later-born neuronal subtypes and glia has provided insight into the temporal program. Early-born neurons, throughout the nervous system, express TFs of the Onecut family, irrespective of the dorsal-ventral progenitor domain from which they are generated. A subsequent wave of neurons expresses *Pou2f2* and *Zfhx2*–*4*, and then a wave of *Nfia*/*b*/*x-* and *NeuroD2*-expressing neurons are produced.^5–7,24–26^ During this final wave, progenitors transition to the production of astrocytes and oligodendrocytes, suggesting that the switch from neurogenesis to gliogenesis is part of a unified temporal patterning program.^5,27^

Temporal changes in the identity of neurons and glia are accompanied by changes in gene expression in NPs. In the vertebrate retina, a conserved transcriptional cascade in progenitor cells controls the specification of differentiated cell types.^28^ Similarly, progenitors in the forebrain^10,26,29^ and midbrain^30,31^ are characterized by a temporal sequence of transcriptional states. Across the neuraxis, *Sox9* and *Nfia*/*Nfib* are sequentially induced in progenitors as they switch from generating early-born to later-born neuronal subtypes and glial cells.^32–36^ Sox9 and Nfia/Nfib play a functional role in controlling the transition from neurogenesis to gliogenesis.^32,34,37^ To what extent the progenitor temporal program controls the different waves of neurogenesis and which aspects of this regulation are conserved across the central nervous system (CNS) remains to be determined.

A set of CREs responsible for directing gene expression in NPs has been identified.^38–42^ In contrast to *Drosophila*,^21,43^ most NPs share a common chromatin landscape, with minimal variation in the accessibility of CREs across progenitor types, at the early stages of neural tube patterning, despite the significant functional and gene expression differences. The similarity in the chromatin landscape between spatially distinct NPs appears to rule out a spatiotopic mechanism. An alternative, therefore, is a chronotopic strategy in which TTFs modify chromatin accessibility in progenitors thereby altering the output of STFs and consequently the cell types specified over time.

To test this possibility and to define the gene regulatory logic integrating spatial and temporal patterning in the vertebrate neural tube, we used chromatin accessibility assays and functional perturbations. The analysis revealed a temporal patterning cascade comprising a set of TTFs that are sequentially induced, independently from spatial inputs, and act to modify chromatin accessibility and alter gene expression over time. Thus, by contrast to *Drosophila*, in the vertebrate neural tube, TTFs establish the chromatin context for spatial determinants to specify cell type identity. This defines a mechanism for generating and organizing cell fate diversity in the developing nervous system.

## Results

### A temporal program of chromatin accessibility in neural tube progenitors

**Figure 1:**
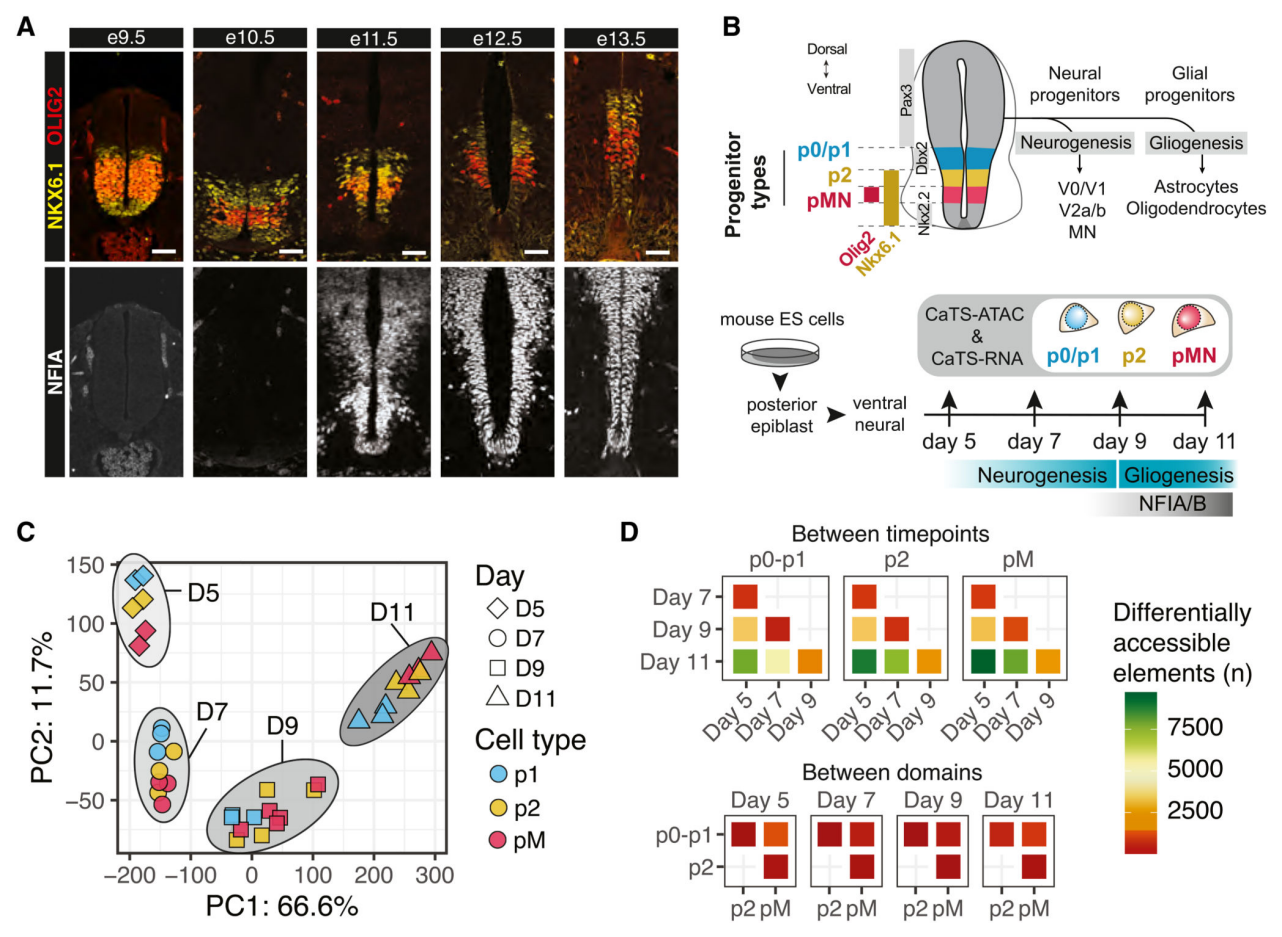
A global chromatin temporal program in spinal cord progenitors (A) Expression of spatial TFs NKX6-1 and OLIG2 in the neural tube across all time points from mouse embryonic day (E) 9.5 to E13.5. The TTF NFIA is first detected at E11.5. Scale bar, 50 μm. (B) Diagram of the neural tube progenitor subtypes characterized in this study and the differentiated cell types they produce (top). Diagram of the *in vitro* system and the time points assayed (bottom). (C) Principal-component analysis (PCA) of CaTS-ATAC for all time points and cell types. The main changes are seen between time points (shapes, and labeled days 5, 7, 9, and 11), for all cell types analyzed (colored). (D) Quantification of the number of differentially accessible regions between time points for each cell type (top) and between cell types for all pairwise cell type comparisons at each time point (bottom) highlights the large number of changes detected over time. See also Figure S1.

The spatially restricted expression of a set of TFs partitions the developing neural tube into discrete domains of progenitors arrayed along the dorsal-ventral axis (Figure 1A).^44^ A distinct set of TTFs defines sequential phases of NP development.^5^ These are expressed in NPs along the entire dorsal-ventral axis (e.g., NFIA; Figure 1A). To understand how NPs integrate their dorsoventral spatial identity with the temporal progression of cell fate changes, we made use of an *in vitro* cellular model based on the directed differentiation of embryonic stem cells (ESCs)^38,45–48^ (Figure 1B). We assayed *in-vitro*-generated ventral NPs from day 5 until day 11 of differentiation. Gene expression recapitulated the temporal hallmarks expected from *in vivo*, including sequential onset of expression of the TFs *Sox9, Nfia*, and *Nfib* (Figure S1A), and the later appearance of glia markers *Slc1a3* (*Glast*) and *Gfap* (Figure S1B). Expression of NFIA at the protein level was detected from day 9, reaching 90% of SOX2+ NPs by day 11 (Figure S1E).

We focused on progenitor cells with three spatial identities, p0–p1, p2, and motor neuron progenitors (pMN). The three progenitor domains generate a variety of temporally distinct neurons (motor neurons [MNs] and interneurons) and later glial cell types (oligodendrocytes and astrocytes).^34,49–55^ This suggests that a temporal program must intersect with the spatial identity of progenitors to determine the fate of progeny. To investigate this, we first performed cell-type-specific assay for transposase-accessible chromatin (ATAC) and RNA sequencing (RNA-seq) using our previously developed method, crosslinked and TF-sorted ATAC-seq (CaTS-ATAC),^38^ separating progenitor cells of either p0–p1, p2, or pMN identity at days 5, 7, 9, and 11. These three progenitor subtypes can be generated in the same signaling conditions and isolated based on a combinatorial code of TF expression: all express SOX2, but pMN cells express OLIG2 and NKX6-1; p2 cells express NKX6-1 only; and p0-p1 do not express either of these TFs (Figures S1C and S1D). In these conditions, there is little or no induction of more dorsal (PAX3) or more ventral (NKX2-2) progenitors (Figures S1F and S1G). Expression of key molecular markers of both temporal (*Sox9, Nfia*, and *Nfib*), and STFs showed the expected patterns for all three progrenitors over time (Figure S1H).

**Figure 2:**
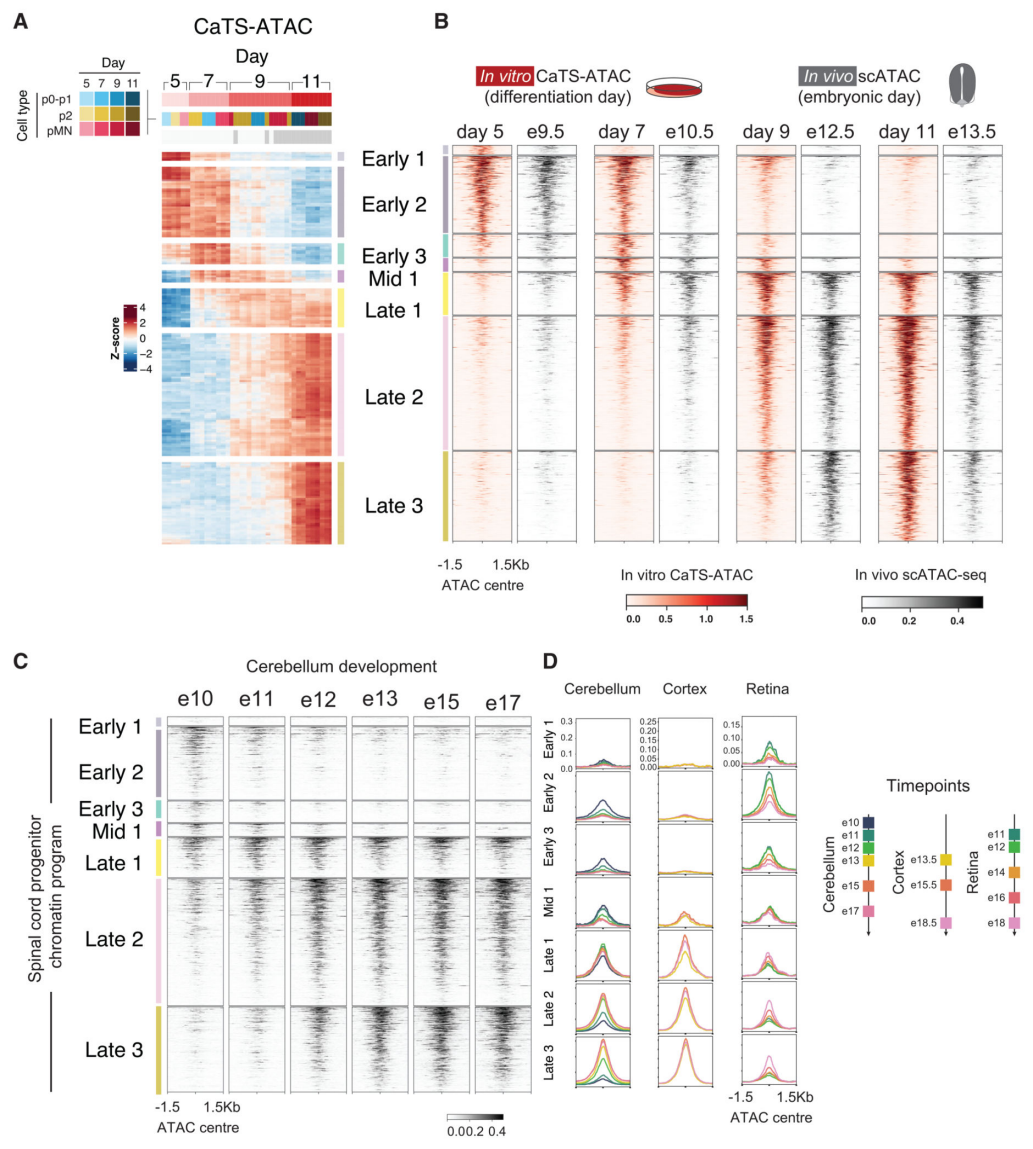
The chromatin temporal program is conserved across the CNS (A) Heatmap of high confidence differentially accessible elements over time, shared in all protenitor cell types using our cellular model and the cell-type-specific sorted ATAC-seq (CaTS-ATAC). (B) Comparison of the temporal elements between *in vitro* differentiation (this study) and *in vivo* scATAC-seq.^56^ (C) The same elements display the same temporal ordering of opening and closing in cells from the cerebellum.^57^ (D) Equivalent accessiblity dynamics for these elements was evident in the developing cortex^3^ and retina.^11^ The cerebellum data in (D) is a summary of the data displayed in (C). See also Figure S2.

Consistent with our previous study, the chromatin accessibility was similar in the three progenitor domains at day 5 (Figure 1C).^38^ Moreover, the three progenitor types had similar chromatin accessibility profiles at each time point. However, the set of accessible regions differed between time points (Figures 1C and 1D). A clear, strong, global temporal signature of chromatin accessibility dominates the chromatin landscape (Figure 2A). Consequently, the number of differentially accessible elements in the same cell type at different time points is far greater than the differences in chromatin accessibility between cell types at the same time point (Figure 1D). Taken together, the data show a strong temporal program of chromatin accessibility that is shared between all three progenitor domains examined.

### A global temporal chromatin program operates *in vivo* throughout the neuraxis

We next asked whether the temporal differences in chromatin accessibility observed *in vitro* were also present *in vivo* and extend beyond the spinal cord to the rest of the developing nervous system. Analysis of *in vivo* spinal cord single-cell ATAC-seq (scATAC-seq)^56^ showed similar temporal chromatin accessibility changes in NPs as observed *in vitro*. Early chromatin elements were accessible at embryonic day (E)9.5 *in vivo*, while late chromatin elements opened at later time points (Figures 2A, 2B, and S2B). The presence of progenitors from across the dorsoventral axis at each time point highlighted that the temporal chromatin program operates across spatial domains *in vivo*. This indicates that *in vitro* differentiation accurately recapitulates *in vivo* developmental transitions and suggests a consistent progression of temporal transitions in chromatin accessibility between *in vitro* and *in vivo* conditions.

Previous studies of more rostral regions of the developing nervous system have identified chromatin elements differentially accessible in early or late progenitors.^11,57^ We therefore examined whether the chromatin program identified in the spinal cord is conserved across the rostral-caudal axis. In the cerebellum,^57^ early temporal elements were open at E10–E11 but closed at later time points, while late temporal elements progressively opened between E11 and E13 (Figures 2C, 2D, and S2C). Similar results were observed in the retina^11^ (Figures 2D and S2E). Data from cortical cells, starting at E13.5,^3^ showed late elements were open (Figures 2D and S2D). Moreover, region-specific reanalysis of an organogenesis atlas^58^ showed that early elements were open at E10.5–E11.5 in di-telencephalon, mesencephalon, hindbrain, and spinal cord cells (Figures S2A and S2F) before closing and late elements opening.

These findings demonstrate that core elements of the temporal chromatin program identified in the developing spinal cord operate globally across CNS regions, although region-specific temporal elements may further complement this conserved program. This raises the possibility that the same TFs act via identical regulatory regions, control chromatin accessiblity across the neuraxis to coordinate temporal progression. Moreover, previous comparative analyses^11,57,59^ suggest that this regulation is likely to be conserved across species, including humans.

### A screen identifies regulators of progenitor temporal progression

We hypothesized that transcriptional factors with temporally changing expression could drive the changes in chromatin accessibility, NPs of different spatial domains share a temporal transcriptional program that is conserved across the rostralcaudal axis.^5^ This program includes around 30 TFs, the expression of which defines sequential developmental phases.^5^ Our CaTS-RNA data recapitulated this sequence: early expression of *Lin28a*/*Lin28b* and *Nr6a1* was followed by expression of *Npas3* and *Sox9* and then later expression of *Nfia/Nfib* (Figure S1I; Table S3).^10^

**Figure 3:**
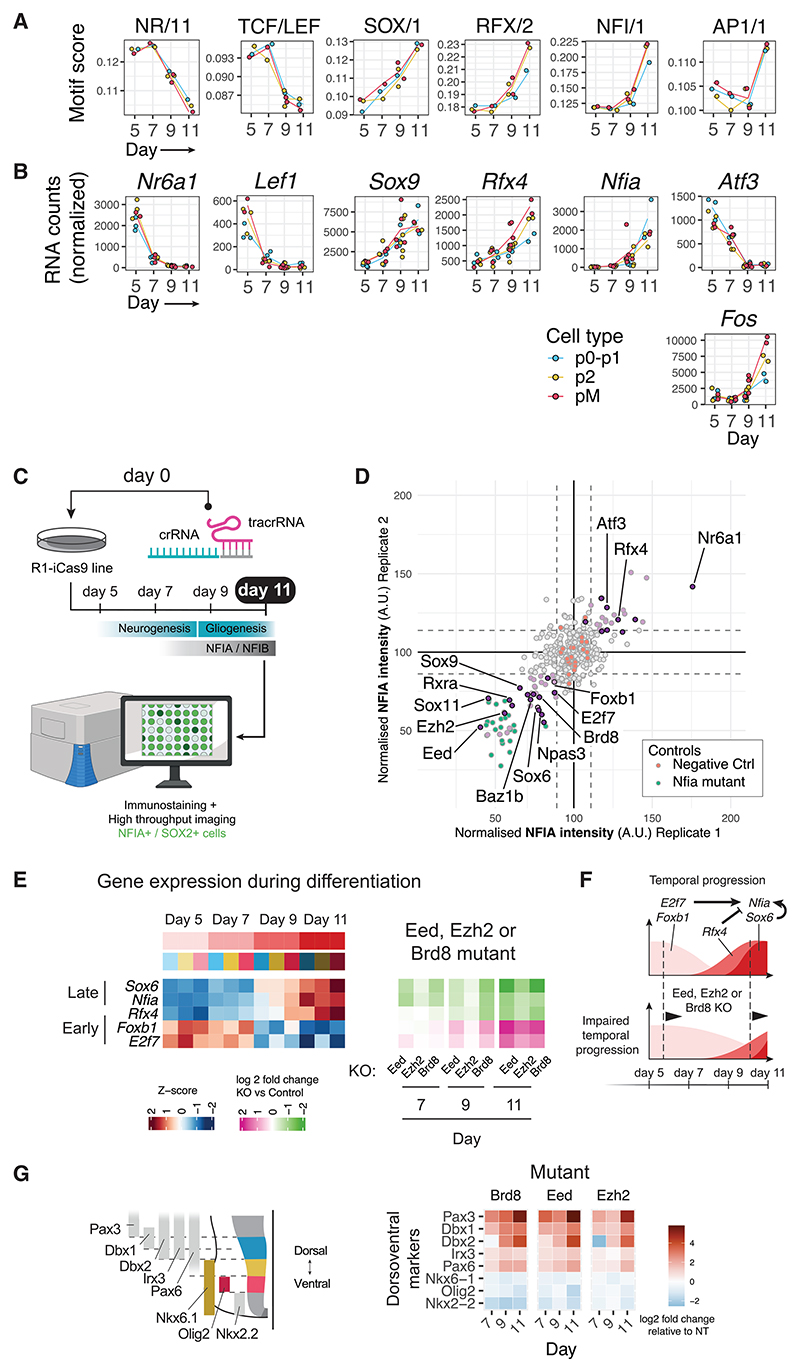
A CRISPR screen identifies regulators of the global temporal program (A) Motifs with differential footprints over the temporal progression. Dots are individual measurements from independent differentiations, lines represent the mean value. (B) Expression levels of TFs with similar temporal dynamics that could bind those motifs. Both dynamic footprints and gene expression occur in a coordinated manner. (C) Diagram of the CRISPR screen setup and readout. (D) Results across two biological replicates (*x* and *y* axis) with controls and some hits highlighted. Negative controls (non-targeting and non-transfected) are in pink, postivite control (Nfia targeted), in green. Solid lines represent the mean value of the negative control in each replicate (normalized to 100), dashed lines show the mean ± 1 standard deviation for each replicate. Hits are labeled in light purple (> or <mean ± 1 SD in both replicates), highlighted gene names are in dark purple. (E) Average expression (CaTS-RNA) of the indicated genes across cell types and time points (left). Fold change expression upon mutation of *Eed, Ezh2*, or *Brd8* for the same genes at three time points. (F) Diagram summarizing the effects of *Eed, Ezh2*, and *Brd8* on the temporal program. Late genes are downregulated, and earlier genes upregulated, consistent with a delayed temporal progression. (G) Diagram of the expression of spatial TFs (left). Dorsal genes are upregulated when *Ezh2, Eed*, or *Brd8* are mutated compared with non-targeting controls. See also Figure S3.

To explore the involvement of temporal transcriptional factors in the changes in chromatin accessibility, we examined differences in motif usage between different times using footprinting to identify motifs likely to be bound by proteins. We used TOBIAS^60^ and grouped footprint-containing motifs into archetypes based on position weight matrix (PWM) clustering.^61^ The analysis revealed several motifs that were predicted to be bound differentially between time points and correlated with changes in gene expression for TFs that bind to these motifs. For example, the expression of *Rfx4* correlated with footprints on an RFX motif (Figures 3A and 3B). At later time points, we see the expected expression and footprint for NFIA TFs. *Sox9* is known to be upregulated at an intermediate stage of NP development^32^ and indeed its expression correlated with the presence of a SOX footprint. However, several other SOX TFs are expressed across this temporal transition and could contribute to the observed footprint (Figure S1J). We also identified the AP1 motif as differentially bound and several TFs that bind this motif are differentially expressed over time (*Atf3, Fos*, and *Junb*). These could individually or in combination explain the observed footprint (Figures 3A and 3B).

These analyses highlighted candidate TFs that may drive the temporal program in NPs. However, a single footprint can result from the effects of multiple TFs and not all TFs produce identifiable footprints. Moreover, many components of chromatinmodifying complexes are differentially expressed over time but are not associated with specific genomic motifs. Therefore, to define drivers of the temporal program, we performed an arrayed CRISPR mutation screen. We selected ~300 differentially ex-pressed genes during the temporal progression, including TFs, chromatin modifiers, and remodelers and members of signaling pathways. We induced Cas9 expression and transfected crRNA: acrRNA at the onset of differentiation. We then assayed the intensity by immunostaining of the late TTF NFIA within SOX2+ regions at day 11 of differentiation. The aim was to identify regulators of the temporal progression that would accelerate or delay the temporal program as measured by an increase or decrease of late NPs (SOX2+ NFIA+) relative to our negative controls (non-targeting crRNAs and non-transfected). As a positive control, we targeted *Nfia* itself (Figure 3C).

The screen identified several positive regulators of NFIA, the targeting of which led to a depletion in NFIA levels, similar to targeting *Nfia* itself (Figures 3D, S3A, and S3B). This included *Sox9*, which is known to be required for the temporal program and the expression of NFIA *in vivo*.^62^ In addition, two subunits of PRC2 (Eed and Ezh2) and two memt--bers of chromatin remodeling complexes (Baz1b and Brd8r-) were identified to be involved in NFIA induction. Conversely, targeting of *Rfx4* and *Nr6a1*, both of which are expressed in NPs earlier than NFIA although starting at different time points, resulted in increased NFIA expression (Figure 3D). This suggests that expression of these factors might normally delay NFIA induction.

We first investigated the effects of *Eed* and *Ezh2*, which are members of the polycomb complex, and *Brd8*. To distinguish whether the reduction in NFIA expression observed in the primary screen was due to lower levels of NFIA expression or a lower proportion of cells expressing NFIA, we performed intracellular flow cytometry for NFIA and SOX2. This revealed that mutation of either *Eed, Ezh2*, or *Brd8* resulted in lower proportion of NFIA+ progenitors, from the onset of NFIA expression at day 9 (Figure S3C). Because the polycomb complex and chromatin remodelers regulate many aspects of cell identity, we performed RNA sequencing (RNA-seq). In agreement with Eed, Ezh2, and Brd8 promoting NFIA expression, NFIA expression was reduced at days 9 and 11 after targeting *Eed, Ezh2*, or *Brd8*, compared to control. Consistent with an overall disruption to the temporal program, genes normally expressed during the early phases (e.g., *Foxb1*) were upregulated at later times and genes normally expressed at late times (e.g., *Rfx4* and *Sox6*) were downregulated (Figures 3E and S3D). These included several of the TFs identified as hits in the screen (Figure 3E). These results suggest a role for the polycomb complex and chromatin remodelers in the progression of the temporal program in the neural tube (Figure 3F). This is consistent with the extension of the early temporal competence window in *Drosophila* neuroblasts lacking PRC1 or PRC2.^63^ Similarly, the polycomb complex has been implicated in controlling neurogenesis^64^ and the neurogenic to gliogenic transition in vertebrates,^65^ although results in the mammalian cortex^10,66^ highlight the complexity of regulating the temporal transitions.

However, mutation of *Eed, Ezh2*, or *Brd8* also altered expression of dorsoventral identity genes, increasing expression of dorsal markers such as *Pax3, Dbx1*, and *Dbx2* (Figure 3G), while the expression of the expected ventral markers, such as *Olig2*, were abrogated (Figures 3G and S3E). These data are consistent with polycomb group proteins and Brd8 concomitantly affecting the temporal progression and spatial identity of NPs.

**Figure 4:**
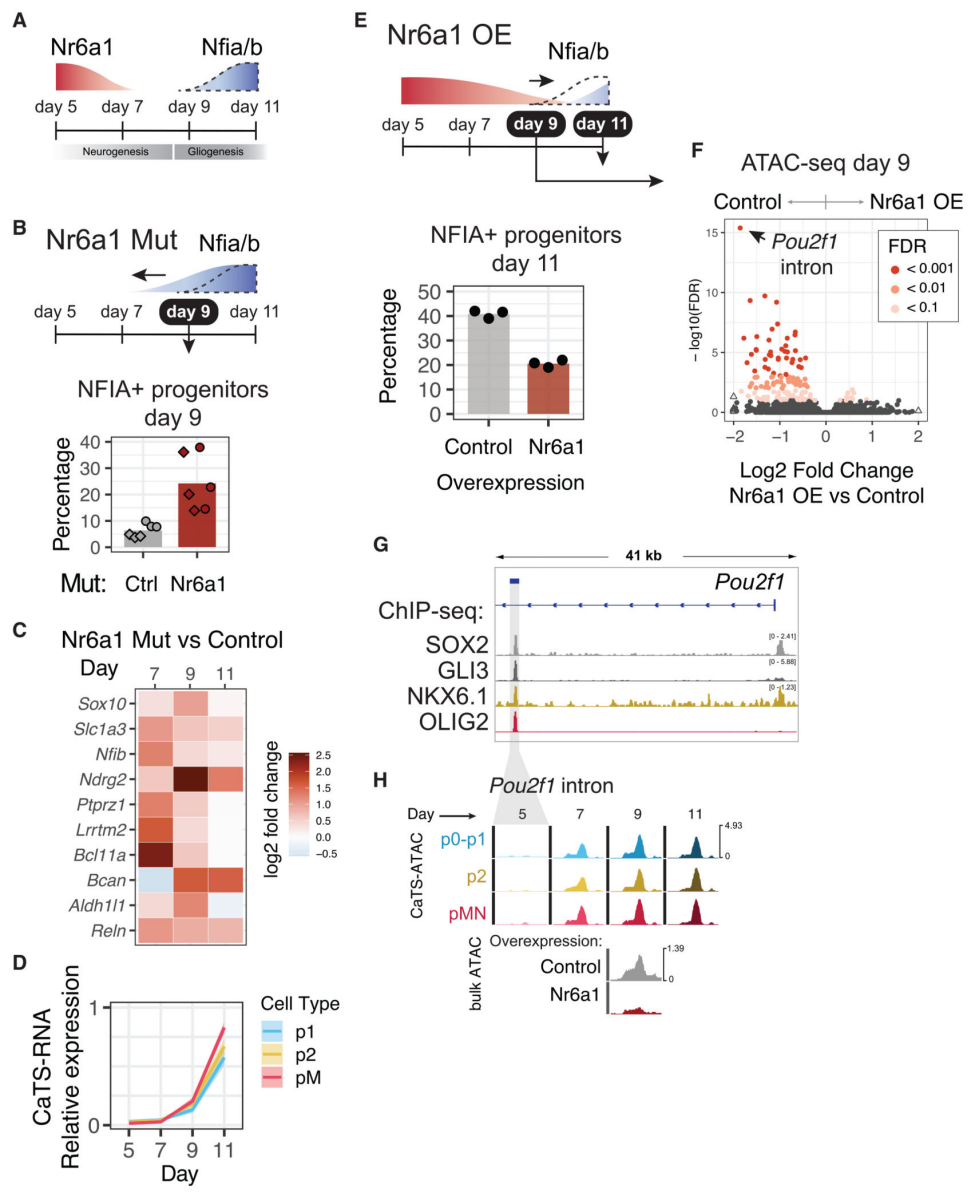
Nr6a1 is a regulator of the temporal program (A) Diagram of the temporal expression of Nr6a1 and Nfia/Nfib. (B) Nr6a1 mutation results in a higher proportion and earlier generation of NFIA+ progenitor cells. Dots are individual measurements, bars represent the mean value. (C) Fold change in Nr6a1 Mutant versus non-targeted cells shows early upregulation of these genes. (D) Average expression of genes in (C) would usually start at day 9 or 11. Lines represent mean relative expression, shaded area is standard error of the mean. (E) Nr6a1 overexpression by lentiviral transduction at day 5 results in reduced proportion of NFIA+ progenitors are day 11. Dots are individual measurements, bars represent the mean value. (F) ATAC-seq at day 9 shows that elements that would have usually opened by day 7 or 9 (Figure S4B) are open in control but not in Nr6a1 overexpression. (G) Image from the genome browser of the Pou2f1 locus showing an element bound by SOX2, GLI, and STF NKX6-1 and OLIG2. (H) CaTS-ATAC of the element from (G) shows the opening of the element in all cell types by day 7 (top). The element opens in control but fails to open after Nr6a1 overexpression (bottom). See also Figure S4.

The primary screen identified orphan nuclear receptor Nr6a1 as a strong negative regulator of NFIA. Both *in vivo* and our CaTS-RNA data show *Nr6a1* is expressed at early time points and sharply dowregulated by day 7 of differentiation (Figures 3A and 4A).^5^ We therefore wanted to test whether *Nr6a1* played a functional role in the control of the temporal dynamics that we have observed. Targeting *Nr6a1* by CRISPR guide transfection resulted in a higher proportion of NFIA-positive progenitors as early as day 9, confirming *Nr6a1* as a negative regulator of NFIA (Figure 4B). Consistent with a disruption of the global program, several genes usually induced at day 9 or later (Figure 4D) were upregulated as early as day 7 (Figures 4C and S4A). These included genes known to be part of the gliogenic switch or implicated in gliogenesis such as *Nfib* itself, *Sox10, Aldh1l1, Bcl11a, Bcan*, and *Slc1a3* (also known as *Glast*) (Figure 4C). This supports a role of *Nr6a1* as a negative regulator of the temporal program.

We tested whether the reverse, overexpression of *Nr6a1*, would delay temporal progression. To prolong the expression of *Nr6a1* beyond its endogenous window, we used lentivirus expression to transduce differentiating NPs at day 5. Flow cytometry analysis at day 11 showed that the proportion of NFIA-positive progenitors was reduced by 50% in *Nr6a1* overexpression versus control (Figure 4E). This is consistent with Nr6a1 maintaining cells in an earlier temporal state and acting as a negative regulator of temporal progression.

We reasoned whether *Nr6a1* is controlling the temporal program via changes in chromatin accessibility, we would expect to see changes in accessibility upon *Nr6a1* overexpression. Consistent with this, ATAC-seq at day 9 identified elements that specifically opened in control, but not in cells overexpressing *Nr6a1* (Figure 4F). These elements correspond to dynamic sites that are usually opened by day 7 or 9 in all cell types (Figure S4B). This included an element near *Pou2f1* (Figures 4G and 4H). Pou2f1 is differentially expressed and has been reported to control temporal progression in *Drosophila* neuroblasts and vertebrate retina.^23,25^

Together, these data support a role for *Nr6a1* in promoting the early chromatin program and antagonizing the temporal progression of NPs.

### The global chromatin temporal program directs position-specific cell type gene expression

**Figure 5:**
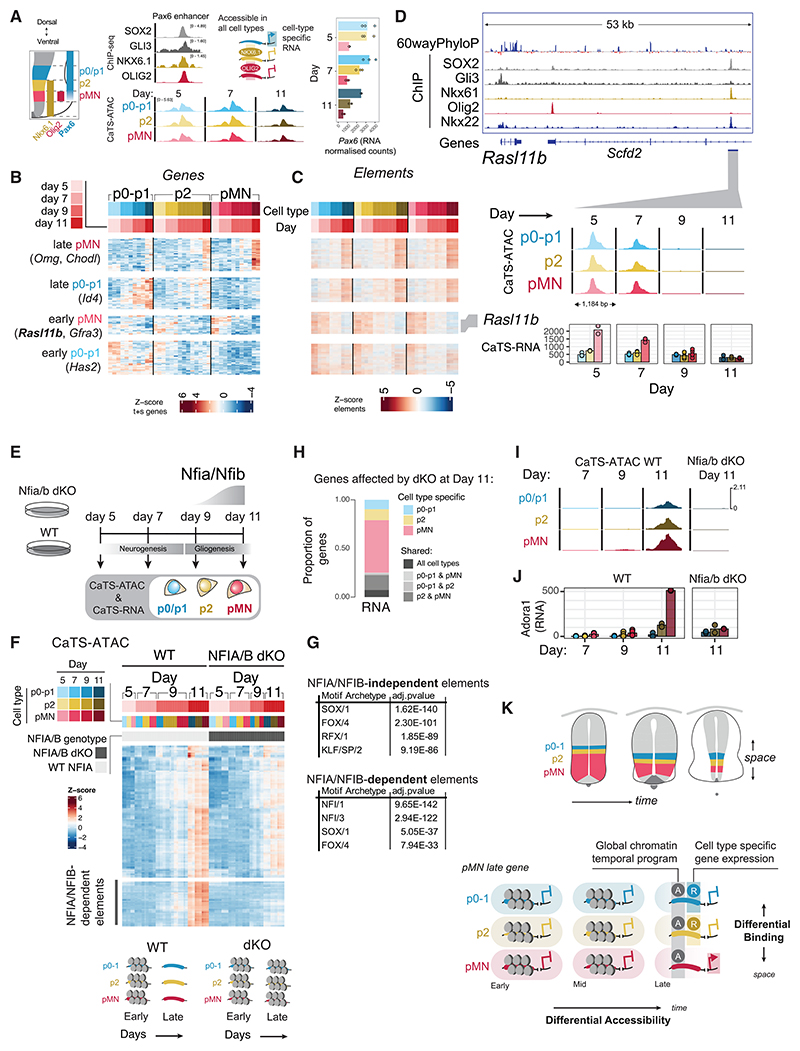
The temporal chromatin program controls dynamic, cell-type-specific gene expression (A) Spatial TF PAX6 is differentially expressed between p0-p1, p2, and pMN (diagram: left, CaTS-RNA: right). A published enhancer element for *Pax6*^40^ is bound by STF NKX6.1 and OLIG2, as well as positive input SOX2 and the Sonic Hedgehog (Shh) effector GLI. The element is open across all cell types and all time points. For *Pax6* RNA expression, dots are individual measurements, bars represent the mean value. (B) Genes dynamically expressed in a temporal and cell-type-specific manner (for full set, see Figures S5B and S5C). (C) Elements associated with genes in (B) show temporally dynamic accessibility that is shared across all cell types. (D) Example element associated with *Rasl11b* is bound by STF NKX6-1 (top) and accessible at days 5 and 7 but not later time points. *Rasl11b* is differentially expressed between cell types. Dots are individual measurements, bars represent the mean value. (E) Diagram of the *Nfia/Nfib* double KO profiling by CaTS-ATAC and RNA. (F) A subset of elements that should open at later time points (25%, 1,834 out of 7,206) fails to open in *Nfia/Nfib* dKO (adjusted *p* < 0.01, log_2_ fold change > 2 and basemean > 100). Affected elements are shared for all cell types. (G) Motif enrichment analysis for elements differentially accessible in Nfia/Nfiab double KO (NFIA/NFIB-dependent) shows NFIA/B motifs are the most significant, supporting a direct role of these factors in the accessibility changes. (H) Breakdown of genes differentially expressed between control and dKO at day 11 showing proportion of shared versus cell-type-specific highlights that genes are primarily affected only in one cell type. (I) Example element associated with *Adora1*, which opens at day 11 but fails to open in the dKO in all cell types. Dots are individual measurements, bars represent the mean value. (J) *Adora1* is a cell-type-specific gene (pMN) expressed at day 11. The dKO fails to express this gene in pMN (cell-type-specific gene effect). (K) Diagram explaining how the temporal and spatial axis integrate time and space via two distinct chromatin strategies. See also Figure S5.

We next asked how differential gene expression between spatial domains was controlled during the temporal progression, given that all spatial domains shared a global temporal chromatin program. Our previous work demonstrated that differential binding of cell-type-specific TFs at commonly accessible elements controls cell-type-specific gene expression in early NPs. This also appears to be the case at later time points. For example, chromatin accessibility associated with *Pax6* (Figure 5A) or *Dbx1* (Figure S5A) is similar in p0–p1, p2, and pMN progenitors at days 5, 7, 9, and 11, despite these genes having distinct spatial expression patterns. A regulatory element documented to control *Pax6* in NPs^40^ is accessible in all cell types and remains accessible over time. The element is bound by NKX6.1 and OLIG2, which are themselves differentially spatially expressed transcriptional repressors. Thus, NKX6.1 binding in p2 and/or pMN, and OLIG2 binding in pMN can repress the *Pax6* expression in these cell types.

We reasoned that differential binding within the temporally available elements could explain the integration of the temporal and spatial patterning programs. We identified groups of genes that were differentially expressed between cell types and between time points (Figure S5B; Table S4). For example, chromatin regions associated with genes expressed at late times but only in pMN (e.g., *Omg*) or only in p0-p1 (e.g., Id4) (Figure 5B) become accessible only at late time points, following the genes’ expression dynamics. However, these regions were accessible in all cell types and not exclusively in the cell type in which the associated gene was expressed (Figures 5C and S5C). This pattern is true for genes displaying other temporal dynamics, such as *Rasl11b*, which is differentially expressed in pMN at early time points but not at later times. Its gene expression profile correlated with chromatin accessibility at a nearby element, which is accessible in early p0-p1, and p2 progenitors as well as pMN cells. This element is bound by cell-type-specific TFs, such as NKX6.1 (Figure 5D), which might confer the spatial specificity to *Rasl11b* from shared chromatin accessibility. This supports a model in which the temporal chromatin program directs the output of the spatial gene regulatory network to achieve dynamic and cell-type-specific gene expression.

To test this model, we perturbed the well-studied regulators of the temporal transition, NFIA and NFIB. *Nfia* and *Nfib* are dynamically regulated over time, with onset at day 9 and peaking at day 11, which is consistent with the appearance of their footprints (Figure 3A). As expected, cells lacking NFIA/NFIB had defects in generating glial cells during *in vitro* differentiation (Figure S5D). If NFIA/NFIB regulate the temporal program, we would predict shared chromatin accessibility changes across all domains. Additionally, if these temporal elements control cell-type-specific genes, we predicted that depletion of NFIA/B would show cell-type-specific defects in gene expression.

We assayed *Nfia*/*Nfib* double knockout (dKO) cell lines across the time course (Figure 5E). This revealed a loss of chromatin accessibility in a substantial number of regions that usually become accessible at day 11. Moreover, most of the affected accessible elements were shared between progenitor cell types (Figure 5F). NFI motifs are the most enriched among the elements affected in the dKO, supporting a direct role of NFIA/ NFIB in opening these elements (Figure 5G). This is consistent with a role for NFIA/NFIB in the temporal program. By contrast, the gene expression changes that resulted from the loss of NFIA/NFIB were mostly cell type specific (Figure 5H). This is also highlighted by examination of specific element-gene pairs. For example, the predicted element associated with *Adora1* gains accessibility at day 11 in all cell types in the wild type but fails to open in *Nfia/Nfib* dKO cells (Figure 5I). *Adora1* is a celltype-specific gene expressed in pMN at day 11 but fails to be expressed in pMNs in dKO cells (Figure 5J). Globally, genes that are affected in a cell-type-specific way in *Nfia/Nfib* dKO cells show reduced chromatin accessibility in their associated elements (in all cell types) (Figure S5E). This indicates that this differentially expressed gene is controlled by the shared chromatin temporal program.

Overall, the data support a model in which the temporal chromatin program is encoded by differential accessibility of elements over time, with specific sets of elements available at each time window. Within these sets of elements, differential binding of TFs converts the shared accessibility to cell-typespecific gene expression. This highlights how the two patterning axes, time and space, are encoded by different chromatin strategies, differential accessibility and differential binding (Figure 5K). This suggests a logic to the integration of the regulatory information.

### The progenitor temporal program controls progeny cell type identity

**Figure 6:**
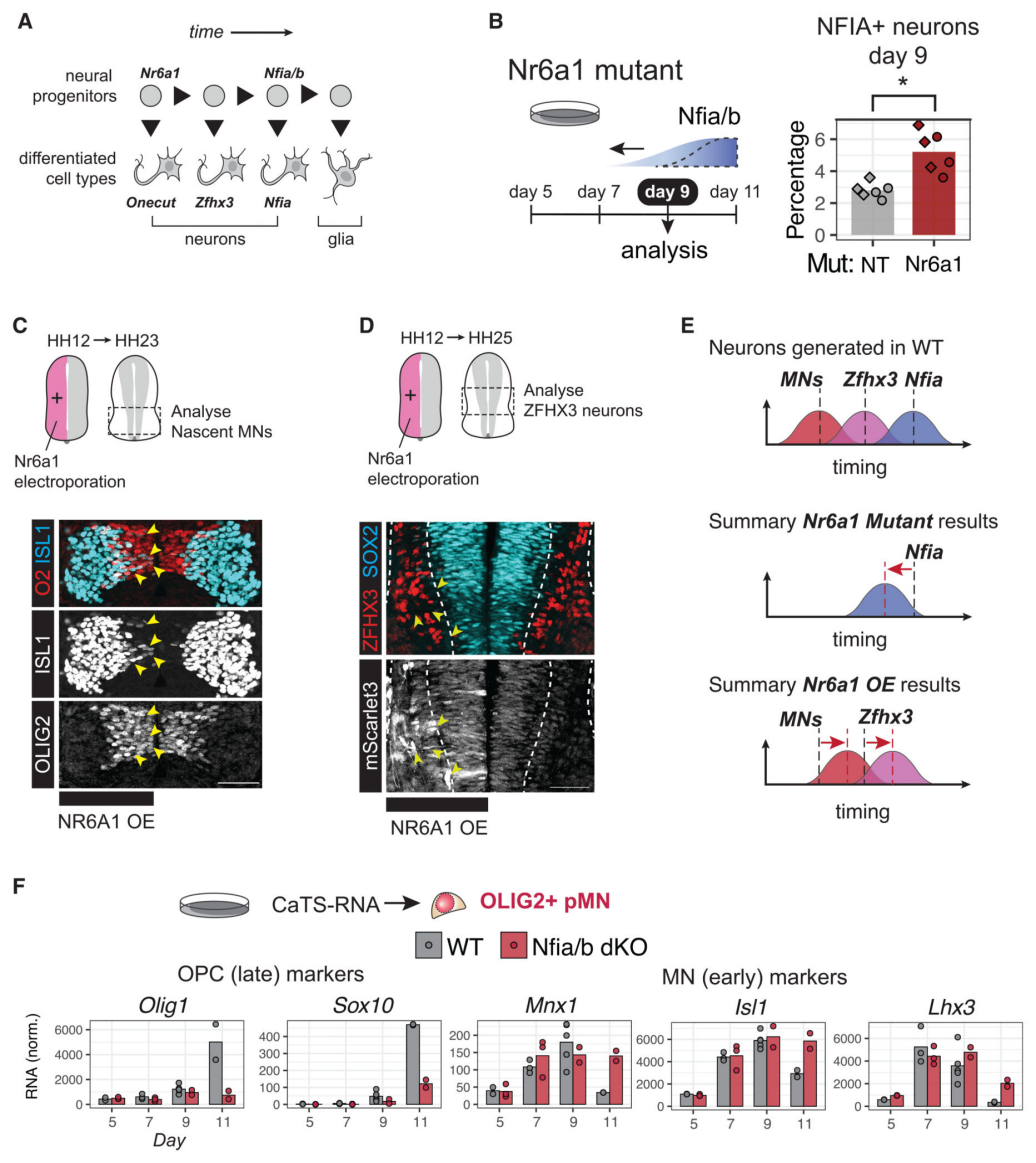
The progenitor temporal program controls differentiated cell type outcomes (A) Diagram of the neuronal temporal program.^5,6^ (B) *Nr6a1* KO results in a higher proportion and earlier generation of NFIA+ neurons. Dots are individual measurements, bars represent the mean value. *p < 0.05, two-sample t test. (C) Spinal cord from HH23 chicken shows ongoing motoneurongenesis (ISL1+ OLIG2+ cells) in the side of Nr6a1 electroporation, performed at HH12. (D) Spinal cord from HH25 chicken shows reduced expression of intermediate neuronal marker ZFHX3 in cells overexpressing Nr6a1. (E) Diagram summarizing the effects seen at the neuronal level in (B)–(D), with *Nr6a1* mutant accelerating late neuronal generation, whereas *Nr6a1* overexpression prolongs generation of MNs (early) and delays onset of ZFHX3 (intermediate time point). (F) Known markers of oligodendrocyte progenitor cells *Olig1* and *Sox10* are not upregulated to the same level as control in cells lacking Nfia/Nfib. Conversely, expression of earlier MN markers is sustained in the dKO (e.g., *Isl1* and *Mnx1*). Dots are individual measurements, bars represent the mean value. Scale bar, 50 μm.

Previous work has identified that neurons born at different times express different TTFs regardless of spatial identity^5,6^: early-born neurons express *Onecut*, intermediate-born neurons express *Zfhx3*, and later-born neurons express *Nfia*/*Nfib* (Figure 6A). To test whether the temporal program in progenitors determines the temporal identity of differentiated cells, we examined the effect of perturbing the temporal program in progenitors.

Further to the effects seen in progenitors (Figure 4B), *Nr6a1* mutation results in an earlier (day 9) and increased production of NFIA+ (late-born) neurons (Figure 6B). To test the effects on the neuronal program, we electroporated *Nr6a1* in chick embryos at HH12 (before the onset of neurogenesis), targeting NPs. Consistent with *Nr6a1* promoting the earlier program and delaying temporal progression, at HH23-24 MNs continued to be generated from *Nr6a1* overexpressed (OE+) pMN cells. This is 12 h after the usual cessation of MN generation.^67^ This is evident by the presence of ISL1-expressing cells within the ventricular zone, close to the apical surface of the neuroepithelium, in NPs (still expressing SOX2+) co-expressing OLIG2 in the electroporated side of the chick neural tube (Figure 6C arrowheads, average ISL1+OLIG2+ cells: 44.33 in electroporated side, 32.50 in control side, *n* = 6 embryos, paired t test *p* = 0.0355). Moreover, assaying embryos at HH25, the time point when ZFHX3+ neurons (intermediate-born) are first detected, revealed a decrease in ZFHX3+ neurons in cells overexpressing *Nr6a1* (Figure 6D). This is consistent with *Nr6a1* promoting the earlier temporal progenitor program, thus delaying the generation of intermediate-born ZFHX3+ neurons, and supports the conclusion that the progenitor program controls the temporal identity of the neurons generated.

Finally, we examined cell-type-specific expression (CaTS-RNA) of known temporal markers of MNs and oligodendrocyte precursors (OPCs). In *Nfia*/*Nfib* dKO cells, we found not only a failure to upregulate markers of the OPC program (*Olig1* and *Sox10*) but also an extended window of expression for early MN markers (*Mnx1, Isl1*, and *Lhx3*) until day 11 (Figure 6F), consistent with the chick data (Figure 6C). Together, these data show that perturbation of the temporal program in progen-itors changes the differentiated cell type output, consistent with the notion that the global chromatin accessibility controls cell diversity generation in the neural tube.

## Discussion

The formation of a functioning nervous system depends on the generation of the right cells in the right place, at the right time. Here, we define a chronotopic mechanism for the integration of the spatial and temporal cues that control the timing and pattern of cell type specification in the vertebrate neural tube. A global temporal chromatin remodeling program operates along both the dorsal-ventral and rostral-caudal axis of the nervous system to modify chromatin accessibility in NPs. This determines the output of spatially specific gene expression programs to govern the time and position of cell type generation. Disrupting key temporal regulators alters lineage progression and differentiated cell identities, demonstrating that the temporal identity of progenitors directs cell fate decisions, as also shown in other regions of the neuraxis.^11^ Together the data indicate that spatial and temporal patterning mechanisms function via distinct regulatory strategies to enable dynamic and cell-type-specific gene expression over developmental time.

Previous work identified temporal gene expression cascades directing sequential transitions in progenitor states.^5,10,31^ Here, we demonstrate perturbing this temporal program in progenitors affects differentiated cell identities. The early factor, Nr6a1 (Germ cell nuclear factor [GCNF]), impedes the temporal progression of NPs and must be downregulated for the regulatory program to proceed. Extending the expression window of Nr6a1 alters the differentiated cell types generated *in vitro* and *in vivo*. Nr6a1 is also implicated in the temporal program of other developing tissues, controlling the timing of Hox gene activation and rostral-caudal patterning during body axis elongation.^68^ Nr6a1 interacts directly with several chromatin modifiers DNMT3b, and methyl-CpG,^69–71^ suggesting that Nr6a1 acts as a global chromatin epigenetic regulator.

While Nr6a1 promotes the early temporal phase, Sox9 and NFIA/NFIB are important for later temporal phases.^62^ The data show that NFIA/NFIB function via modifying chromatin accessibility and are necessary for the temporal progression of cell type specification. Loss of NFIA/NFIB affected numerous elements in progenitors that are predicted to mediate late gene induction events. The affected genes were spatially variable, which is expected if spatial determinants bind temporally available sites in a cell-type-specific way. NFIA/NFIB and SOX9 regulate chromatin accessibility in several tissues^37,72,73^ and recruit the chromatin remodeling complexes.^73–75^ Whether the temporal expression of chromatin-modifying and -remodeling complexes is a crucial part of the temporal program of the neural tube remains to be determined. The progressive downregulation of PRC2 in mouse cortex has been implicated in temporal progression in the cortex^10^ and has been suggested to be part of an epigenetic barrier to neuronal maturation,^76^ supporting a causal role for epigenetic regulators in controlling timing. In these contexts, PRC2 inhibition results in rapid progenitor progression, with increased cell-cycle exit and shortened neurogenesis, or increased speed of neuronal maturation. These contrast with our observation of impaired progression within the progenitor program and highlights the complex roles that PRC2 has in progenitor self-renewal versus neuronal differentiation and maturation.

The scale of the temporal changes in chromatin accessibility in NPs was unexpected given the similarity of the chromatin landscape between spatially distinct progenitor domains.^38^ The dominance of differential accessibility underpinning temporal changes, compared with spatial patterning, suggests a strategy for the integration of spatial and temporal programs. In this view, temporal factors act similarly to “pioneer factors,”^77^ altering the chromatin landscape and defining the set of available CREs. Eviction of spatial determinants from recently closed elements and their recruitment to newly opened elements would occur in a domain-specific manner due to the spatially restricted expression of the spatial factors. This chronotopic mechanism converts shared accessibility across progenitor domains into spatial heterogeneity in TF binding to determine domain- and time-specific gene expression. Mapping the binding patterns of temporal versus spatial factors across these elements over time and understanding how the accessibility of regulatory elements changes will further define this regulatory logic.

This strategy for integrating spatial and temporal patterning appears distinct from that operating in *Drosophila* neural development, where STFs, expressed early in distinct progenitor cells, establish lineage-specific chromatin accessibility profiles that remain constant over time—a spatiotopic chromatin landscape.^21^ *Drosophila* TTFs thus generate cell type diversity by imparting a different gene expression output in each lineage, depending on the chromatin accessibility established by STF. By contrast, in the vertebrate neural tube, the opposite regulatory logic prevails: vertebrate TTFs sculpt a global temporal chromatin landscape that directs where spatially restricted TFs bind. Changes in the TTFs over time alter chromatin accessibility in all lineages, producing the chronotopic chromatin landscape. For example, TTFs such as NFIA/B establish progressive opening of regulatory elements in all progenitor domains. The differential chromatin accessibility over time results in temporally varying lineage-specific gene expression and cell type generation.

What might account for this difference in regulatory logic between the fly and vertebrate nervous systems? In vertebrates, patterning occurs as the neural tube grows and the spatial cues, provided by morphogen gradients, act dynamically.^1^ Accordingly, progenitors change their expression of spatial TFs as the neural tube develops. This may favor a strategy in which a global temporal program controlling chromatin landscapes across progenitors provides regulatory elements for morphogen controlled spatial cues to bind differentially over time. By contrast, *Drosophila* neuroblast lineages are established early and maintain their spatial identity throughout development.^21^ Perhaps this favors a strategy in which spatially distinct chromatin landscapes persist to direct differential TTF binding. Overall, the *Drosophila* and vertebrate strategies highlight alternative but effective solutions to a common problem: generating cell type diversity across space and time from multipotent progenitors. Intriguingly, a shared set of temporal elements are used across *Drosophila* leg, wing, and haltere imaginal discs, with different regulators in each appendage differentially interpreting the same CREs.^78^ This suggests that chronotopic integration might be a general mechanism across species.

These results have important implications for engineering specific neuron and glia subtypes using stem cells. Much attention has focused on establishing the appropriate spatial gene expression profile to reprogram cells.^79–81^ Less regard has been given to the temporal program. However, our data indicate that the interpretation of the spatial program depends on the temporal chromatin state. Considering only the spatial transcriptional program risks incomplete or misdirected reprogramming. Inappropriate temporal states may result in required regulatory elements being inaccessible, or inappropriate elements being accessible, thereby misdirecting spatial TFs. This might explain inefficiencies and off-target results of some current directed differentiation protocols. Our results suggest that imposing the desired temporal epigenetic state followed by manipulating spatial identity might allow more rapid generation of clinically relevant cell types, such as oligodendrocyte progenitors. Overall, clarifying the regulatory logic directing progenitor temporal identity will likely improve directed differentiation and reprogramming strategies.

In summary, our study advances understanding of the regulatory logic governing NP diversity throughout the nervous system, revealing a chronotopic strategy for the integration of global temporal and spatial patterning. This mechanism offers a framework to conceptualize and study the molecular basis for cellular complexity of the nervous system.

### Limitations of the study

In this work, we used mouse ESC-derived models of spinal cord NPs. While we demonstrated that the temporal chromatin elements identified exhibit similar dynamics *in vivo* across multiple CNS regions, additional region-specific temporal elements may operate *in vivo* that are not captured in our culture conditions. To characterize the role of Nr6a1, we performed crRNA:tracrRNA transfection, which will result in mutations in a fraction of the differentiating stem cells. The transcriptomic characterization upon Nr6a1 mutation therefore represent a mosaic population. Clonal cell lines with specific mutations might provide further information on the role of Nr6a1. While our analyses identified regions that become differentially accessible over time, we cannot definitively establish causality between TF binding and chromatin opening from our current data. The temporal correlation between TF expression and element accessibility suggests, but does not prove, direct regulatory relationships. Future experiments incorporating more detailed mechanistic studies will be needed to establish direct binding and causation.

## Resource Availability

### Lead contact

Further information and requests for resources and reagents should be directed to and will be fulfilled by the lead contact, James Briscoe (james. briscoe@crick.ac.uk).

### Materials availability

Murine ES cell lines generated in this study area available upon request.

## Star★Methods

### Key Resources Table

**Table:** 

REAGENT or RESOURCE	SOURCE	IDENTIFIER
Antibodies		
Mouse anti-Nkx6.1	DSHB	F55A10
Rabbit anti-Nfia	Atlas Antibodies	Cat#HPA008884
Goat anti-Olig2	R&D Systems	Cat#AF2418
Rabbit anti-Olig2	Merck Millipore	Cat#AB9610
Sheep anti-Zfhx3	R&D Systems	Cat#AF7384
Goat anti-Isl1	R&D Systems	Cat#AF1837
Rat anti-RFP	Chromotek	Cat#5f8
Rabbit anti-Nfia	Atlas Antibodies	Cat#HPA008884
Goat anti-Sox2	R&D Systems	Cat#AF2018
Mouse anti-Sox2	Santa Cruz	Cat#sc-365823
Rabbit anti-Gfap	Agilent (DAKO)	Cat#Z0334
Sox2-V450	BD Biosciences	Cat#561610; clone O30-678
Nkx6.1-PE	BD Biosciences	Cat#563338; clone R11-560
Tubb3-A647	BD Biosciences	Cat#560394
Pax3-APC	R&D Systems	Cat#IC2457A
Nkx2.2-PE	BD Biosciences	Cat#564730; clone 74.5A5
Donkey anti-rabbit AF488	Invitrogen	Cat#A-21206
Donkey anti-goat AF488	Invitrogen	Cat#A-11055
Donkey anti-mouse AF488	Invitrogen	Cat#A-21202
Donkey anti-goat AF647	Invitrogen	Cat#A-21447
Donkey anti-mouse AF647	Invitrogen	Cat#A-31571
Donkey anti-rabbit AF647	Invitrogen	Cat#A-21245
Donkey anti-guinea pig	Invitrogen	Cat#A-21450
Donkey anti-mouse AF568	Invitrogen	Cat#A-10037
Donkey anti-goat AF568	Invitrogen	Cat#A-11057
Donkey anti-rabbit AF568	Invitrogen	Cat#A-11011
Bacterial and virus strains		
MAX Efficiency™ Stbl2™ Competent Cells	Invitrogen	Cat#10268019
Chemicals, peptides, and recombinant proteins		
Dulbecco’s Modified Eagle Medium (DMEM) Knock OUT	Gibco	Cat#10829-018
Advanced DMEM-F12	Gibco	Cat#21331-020
ES Foetal Bovine Serum (FBS)	PAN Biotech	Cat#P30-2602
Penicillin/Streptomycin	Gibco	Cat#15140122
L-Glutamine	Gibco	Cat#25030024
Non-essential Amino Acids	Gibco	Cat#11140-035
2-mercaptoethanol	Gibco	Cat#21985-023
Phosphate Buffer Saline (PBS)	Gibco	Cat#14190-094
Trypsin-EDTA (0.05%)	Gibco	Cat#25300054
Neurobasal Medium	Gibco	Cat#A35829-01
N2 Supplement	Gibco	Cat#17502001
B27 Aupplement	Gibco	Cat#17504001
Bovine Serum Albumin (BSA) Solution	Sigma-Aldrich	Cat#A7979-50ML
Accutase	Gibco	Cat#A1110501
Recombinant Mouse LIF Protein	Sigma-Aldrich	Cat#ESG1107
PD0325901	Cambridge Biosciences	Cat#SM26-2
CHIR99021	Axon Medchem	Cat#1386
bFGF	R&D Systems	Cat#100-18B
Retinoic Acid (RA)	Sigma-Aldrich	Cat#R2625
Smoothened Agonist (SAG)	Calbiochem	Cat#566660-5mg
Doxycycline	Sigma-Aldrich	Cat#D9891
Puromycin	Sigma-Aldrich	Cat#P8833
Matrigel	Corning	Cat#356231
Gelatin	Gibco	Cat#G1393-100ML
Pierce™ 16% Formaldehyde (w/v), Methanol-free (PFA)	Thermo Scientific	Cat#28908
RLT Lysis Buffer	Qiagen	Cat#1015762
LIVE/DEAD™ Fixable Dead Cell Stain Near-IR	Invitrogen	Cat#L34976
DAPI	Sigma-Aldrich	Cat#D9542
AMPureXP Beads	Beckman Coulter	Cat#A63882
RNasin Plus RNase Inhibitor	Promega	Cat#N2615
Glyoxal (40%)	Sigma-Aldrich	Cat#50649-100ML
TRIzol Reagent	Invitrogen	Cat#15596026
RNase-free GlycoBlue	Thermo Scientific	Cat#AM9516
SYBR Green	Thermo Scientific	Cat#S7563
2X PCR Master Mix NEB	NEB	Cat#M0541S
ProLong™ Gold Antifade Mountant	Invitrogen	Cat#P36930
Igepal CA-630	Sigma-Aldrich	Cat#I8896-100ML
Tween-20	Sigma-Aldrich	Cat#P2287-500ML
Triton X-100	Sigma-Aldrich	Cat#T8787
Digitonin	Invitrogen	Cat#BN2006
Tn5 recombinant protein	This study	N/A
X-tremeGENE™ HP DNA Transfection Reagent	Roche	Cat#6366244001
HBSS Buffer	Gibco	Cat#14170-088
siRNA Buffer (5x)	Horizon Discovery	Cat#B-002000-UB-100
Opti-MEM I Reduced Serum Medium	Gibco	Cat#31985062
DharmaFECT 4 Transfection Reagent	Horizon Discovery	Cat#T-2004-01
Critical commercial assays		
Genome-CRISP™ Inducible Cas9 ROSA26 Mouse Safe Harbor Knockin Kit-Puro	GeneCopoeia	Cat#SH066
RNeasy Mini Kit with DNAse digest	Qiagen	Cat#74106
SuperScript™ III First-Strand Synthesis System	Invitrogen	Cat#18080-051
PowerUp™ SYBR™ Green Master Mix	Thermo Scientific	Cat#A25742
DNA Clean & Concentrator-5 Kit	Zymo Research	Cat#D4013
Dead Cell Removal Kit	Miltenyi Biotec	Cat#130-090-101
SMART-Seq HT Kit	Takara	Cat#634437
Nextera XT DNA Library Preparation Kit	Illumina	Cat#FC-131-1096
NEBNext Ultra II Directional Kit	NEB	Cat#E7760L
QIAseq FastSelect -rRNA HMR Kit	Qiagen	Cat#334386
QIAGEN Plasmid Plus Maxi Kit	Qiagen	Cat#12963
Phusion Flash High-Fidelity PCR Master Mix	Thermo Scientific	Cat#F548L
DNA High Sensitivity DNA Kit	Agilent	Cat#5067-4626
Deposited data		
CaTS-ATAC, CaTS-RNA-seq, bulkATAC-seq, bulk RNA-seq	This study	GSE264172
Experimental models: Cell lines		
*Mus musculus* (Male): HM1	Magin et al.^82^	N/A
*Mus musculus* (Male): HM1 Nfia;Nfib knockout mutant (dKO)	This study	N/A
*Mus musculus*: R1 embryonic stem cell line	ATCC	Cat#SCRC-1011
*Mus musculus*: R1-iCas9 embryonic stem cell line	This study	N/A
*Human*: HEK293T	ATCC	Cat#CRL-3216
Experimental models: Organisms/strains		
Mouse: CBA mice	Charles River	Strain code: 609
Mouse: C57BL/6J mice	Jackson Laboratory	RRID:IMSR_JAX:000664
Mouse: Outbred UKCrl:CD1 (ICR) mice	Charles River	Strain code: 022
Fertilized chicken embryos (Gallus gallus)	Henry Stewart & Co. Ltd	N/A
Oligonucleotides		
qPCR primers used in this study, see Table S1	This study	N/A
Edit-R tracrRNA	Horizon Discovery	Cat#U-002005
Edit-R crRNA Non-targeting Control #1	Horizon Discovery	Cat#U-007501-01-05
Oligonucleotides for bulk ATAC-seq, see Table S1	This study	N/A
Oligonucleotides for CaTS-ATAC and ATAC-seq library, see Table S1	This study	N/A
Recombinant DNA		
SH354-ROSA26 Cas9 knockin donor clone-TRE3G-Puro	GeneCopoeia	Cat#SH354
T2A-mScarlet3 fusion (pmScarlet3_C1)	Addgene	Cat#189753
Ef1a:mScarlet3 expression vector	This study	N/A
Ef1a:Nr6a1-T2A-mScarlet3 expression vector	This study	N/A
Ef1a:mScarlet3 expression lentiviral vector	This study	N/A
Ef1a:Nr6a1-T2A-mScarlet3 expression lentiviral vector	This study	N/A
Software and algorithms		
FlowJo software (v10.8.2)	Becton, Dickinson	https://flowjo.com/flowjo/download
Fiji	Schindelin et al.^83^	https://imagej.net/software/fiji/
R(v4.1.0)	R Core Team	https://www.r-project.org/
nf-core atacseq pipeline (v1.2.1)	https://nf-co.re/atacseq	https://doi.org/10.5281/zenodo.2634132
nf-core rnaseq pipeline (v3.5)	https://nf-co.re/rnaseq	https://doi.org/10.5281/zenodo.1400710
DESeq2 (Version 1.34.0)	Love et al.^84^	https://doi.org/10.18129/B9.bioc.DESeq2
ArchR	Granja et al.^85^	N/A
TOBIAS footprinting tools (Version 0.13.0)	Bentsen et al.^60^	https://doi.org/10.1038/s41467-020-18035-1
AME(v5.1.1)	MEME suite; McLeay and Bailey^92^	http://meme-suite.org/tools/ame
Other		
autoMACS® Pro Separator	Miltenyi Biotec	Cat#130-092-545
SuperFrost Plus™ Adhesion Slides	Thermo Fisher Scientific	Cat#10149870
CellInsight™ CX7 Pro High Content Screening (HCS) Platform	Thermo Fisher Scientific	Cat#HCSDCX7LEDPRO
Flat ^Clear Polystyrene Bottom 96-well Plates	Greiner	Cat#655090
CellBIND 6-well Plate	Corning	Cat#CLS3335
CellBIND 12-well Plate	Corning	Cat#CLS3336
96-well Clear V-Bottom Polystyrene Not Treated Microplate	Corning	Cat#3897
1.5 ml DNA LoBind® Tubes	Eppendorf	Cat#022431021
Phasemaker Tubes	Invitrogen	Cat#A33248

## Experimental Model And Study Participant Details

### Mice maintenance and husbandry

Mice of the following F1(B6xCBA), C57BL6 and outbred UKCrl:CD1 (ICR) (Charles River) mice were used for timed matings. Embryos for analyses were collected at the indicated time points following a mating, with the day of plug detection designated E0.5.

All animal procedures were carried out in accordance with the Animal (Scientific Procedures) Act 1986 under the Home Office project licence PP8527846. Animals were housed under a 12-h light, 12-h dark cycle at the Francis Crick Institute animal facility. Animals were housed in singly-ventilated cages. The relative humidity was kept at 45 to 65%. Mouse rooms and cages were kept at a temperature range of 20-24°C. Animals had 24 hour access to RO water and autoclaved pelleted food. Caging, bedding, nesting materials and enrichments were autoclaved prior to use and cages were changed routinely. A maximum of 5 adult mice were housed per cage if they were over 20 g. Animals were monitored visually daily for health concerns and once a week a full health check was carried out during a discretionary cage change.

### Chicken embryos

Fertilized chicken eggs (Gallus gallus) were obtained from Henry Stewart & Co. Ltd and were never incubated beyond two thirds of development.

### Cell lines

Wild-type experiments were performed with the mouse embryonic stem cell line HM1^82^ (Thermo Fisher, MES4303). The Nfia;Nfib knockout mutant mouse ES cell line (dKO) was generated into the HM1 wild-type background.^5^ All cell lines were maintained at 37°C with 5% carbon dioxide (CO2).

### Generation of R1-iCas9 ES cell line

R1-iCas9 (Clone 2E9) mouse embryonic stem cell line was generated using a modified version of the Genome-CRISPTM Mouse Rosa26 Safe Harbour Gene Knock-in Kit and SH354-ROSA26 plasmid (GeneCopoeia). A Cas9 expression cassette was cloned into a FLAG tagged SH354 plasmid with a Tet-On 3G promoter system (driven by EF1a) integrated into the Rosa26 safe harbour locus in the R1 mouse embryonic stem cell line (ATCC® SCRC-1011TM Positive clones were selected using 0.5ug/ml Puromycin, followed by single cell cloning. R1-iCas9 cells were initially cultured in 2i condition (N2B27 medium supplemented with 1 μM PD0325901 (Cambridge Biosciences), 3 μM CHIR99021 (Axon) and 10 ng/ml mouse LIF (BioLegend)). Cells were adapted to grow on feeders by passaging them for five times.

## Method Details

### Cell culture and neural progenitor differentiation

Mouse ES cells were maintained on a ‘feeder’ layer of mitotically inactivated mouse embryonic fibroblasts (MEFs, derived and expanded in-house) in ES cell medium (Dulbecco’s Modified Eagle Medium (DMEM) Knock Out (Gibco; 10829-018) supplemented with 10% Foetal Bovine Serum (Pan Biotech; P30-2602), Penicillin/Streptomycin (Gibco; 15140122), 2 mM L-Glutamine (Gibco; 25030024), 2 mM Non-essential amino acids (Gibco, Cat No. 11140-035), and 0.1 mM 2-mercaptoethanol (Gibco; 21985-023)) with 1000 U/ml LIF (Chemicon, Int ESG1107). ES cell medium was changed every day. Cells were grown on 6-well plates or p6 dishes and passaged every second day. ES cells were washed once with PBS (Gibco; 14190-094) and then trypsinized using 0.05% Trypsin-EDTA (Gibco, 25300054) for 3 min at 37°C, spun down, resuspended in ES cell medium and 300,000 cells in 1.5 ml were seeded per well (500,000 in 4 ml per 1x p6).

One day before differentiation, CellBIND 6-well dishes (Corning) were coated with 0.1% gelatin (Sigma) overnight at 37°C. On Day 0 (D0, D – differentiation day) ES cells were washed once with PBS and dissociated using 0.05% Trypsin-EDTA (Gibco; 25300054) for 4 min at 37°C. Cells were resuspended in 10 ml ES media. For differentiation of ES cells, feeders were removed (‘panning’) by incubating ES cell + feeder suspension for 20 min at 37°C on 0.1% gelatin-coated 10 cm plates. This process was repeated once. For 11-day differentiations, 60-80,000 cells were plated onto the pre-coated CellBIND dishes (Corning) in N2B27 medium (Advanced DMEM - F12 (Gibco; 21331-020) and Neurobasal medium (Gibco; A35829-01) (1:1), supplemented with 1xN2 (Gibco; 17502001), 1xB27 (Gibco; 17504001), 2 mM L-glutamine (Gibco; 25030024), 40 μg/ml BSA (Sigma-Aldrich, Cat No. A7979-50ML), and 0.1 mM 2-mercaptoethanol) supplemented with 10 ng/ml bFGF (R&D; 100-18B). On day 2, the media was changed to N2B27 supplemented with 5 μM CHIR99021 (GSK3β inhibitor; Axon, 1386). 20-21 h later, on day 3, the media was changed to N2B27 with 100 nM RA and 10 nM of Smoothened Agonist (SAG; Calbiochem, 566660). From day 5 onwards, cells were kept in N2B27 supplemented with 10 nM SAG. From day 3 onwards, media changes were performed daily.

The differentiations described as “0 nM SAG” were performed as described above with no addition of SAG.

### Imaging-based CRISPR-Cas9 mutant screen

#### Preparation of custom library

96-well plates containing the crRNA Cherry-Pick Custom Library (0.1 nmol, 5 crRNA pool) from Horizon Discovery were spun down briefly and de-sealed in a Class II Laminar flow hood using aseptic technique. The library includes a pool of 5 crRNAs for each gene of interest per well. 50 μl of 1x siRNA buffer (Horizon Discovery, B-002000-UB-100) per well was added to make up a 2 μM crRNA stock. Plates were covered and rocked gently on a plate shaker for 30 min. Plates were then spun down briefly (1 min, 1000 rpm at room temperature). To prepare libraries, 10 μl of 2 μM crRNA stock was transferred to new V-bottom 96-well plates using a 96-well electronic pipette VIAFLO 96 (Integra Biosciences). 40 μl 1x HBSS buffer was added to make a 400 nM stock. 60 ml of 400 nM tracrRNA was prepared. 50 nmol tracrRNA was resuspended in 500 μl 1x crRNA to make a 100 μM stock. 240 μl of this stock was diluted in 59.76 ml of HBSS buffer. 50 μl of 400 nM tracrRNA was added per well to make 100 μl of 200 nM crRNA:tracrRNA stock. All crRNA and crRNA:tracrRNA stock 96-well plates were sealed and stored at -20°C until further use.

### Screen differentiation

One day before transfection with CRISPR guides, Cas9 was induced in R1-iCas9 ES cells by exposure to 1 μg/ml Doxycycline (Sigma-Aldrich; D9891), which was supplemented to the ES cell medium. On the next day, flat μClear polystyrene bottom 96-well plates (Greiner, PS, F-Bottom, 655090) were coated with 70 μl matrigel (Corning, 356231, 1:50 in DMEM/F12) per well. Plates were briefly spun down for 1 min at 1000 rpm and incubated for 3 h at room temperature. ES cells were prepared as described above. During the panning steps, CRISPR guide RNA (gRNA) complex and transfection reagents were prepared.

The Edit-R CRISPR-Cas9 platform (Horizon Discovery) was used. The platform includes two components required for gene editing: A chemically synthesized trans-activating CRISPR RNA (tracrRNA), and a chemically synthesized CRISPR RNA (crRNA) designed for the gene target site of interest. For transfection, 5 crRNAs (20 μM stock) were pooled and combined with tracrRNA (20 μM stock, U-002005) to form a guide RNA (gRNA) complex (crRNA:tracrRNA) (final concentration: 10 μM). gRNA was then diluted in 1x HBSS buffer (Gibco, 14170-088) to make up 200 nM. As an example: 10 μl crRNA (2 μl of each individual crRNA), 10 μl tracrRNA were combined (20 μl of gRNA). 20 μl of gRNA was diluted into 1 ml HBSS. In parallel, negative, and positive controls were prepared. Non-targeting control (Control #1, 5 nmol stock, Horizon Discovery, U-007501-01-05) was used as negative control. Pool of 5 crRNAs against Nfia was used as positive control.

Matrigel was removed and plates were washed once with 200 μl PBS per well. 10 μl of the gRNA mix was added per well on the coated 96-well plates. 10 μl of diluted transfection reagent (DharmaFECT4, Horizon Discovery, T-2004-01, ~0.15-0.2 μl per 10 μl Opti-MEM I Reduced Serum Medium (Thermo Fisher; 31985062)) was added.

R1-iCas9 cells were counted and seeded in 80 μl N2B27 medium with 10 ng/ml bFGF (R&D; 100-18B) and 1 μg/ml Doxycycline in each well. The final volume per well is 100 μl with a gRNA final concentration of 20 nM. For most experiments, 4500 cells/well were added (for the non-transfected and non-targeting controls 2500 cells/well). Per plate, at least 3 technical replicates for each targeted gene and both controls were included. Plates were rocked side-to-side to distribute cells evenly and left to sit for 5 min. Plates were moved to incubators at 37°C. Gene-edited cells were differentiated according to the previously described *in vitro* protocol.

### Staining and readout

On day 11, plates were washed once with PBS (100 μl/well) and fixed with 4% paraformaldehyde for 15 min at room temperature. PFA was removed and plates were washed once with 200 μl PBS. 60 μl primary antibodies (anti-rabbit NFIA, 1:1000; anti-goat SOX2, 1:500) in blocking solution (PBS, 0.1% BSA and 0.1% Triton X-100) were added to each well and incubated overnight at 4°C. The next day, cells were washed once for 15 min with PBST (PBS supplemented with 0.1% Triton X-100), and secondary antibodies (Alexa 488 anti-rabbit, 1:1000; Alexa 647 anti-goat, 1:1000 in blocking solution) were added. Plates were incubated for 2-3 h at room temperature protected from light. Plates were washed once with PBS and DAPI (1:1000) for 5 min, followed by a second wash for 5 min. 200 μl PBS was added to each well, plates were sealed and stored at 4°C until imaging. Imaging was carried out within a week of staining.

### Image acquisition and processing

Plates were imaged on the CellInsight CX7 Pro HCS Platform (Thermo Fisher) using the HCS Studio V6.6.2 software and analysed using the Cellomics Compartmental Analysis V4 (Thermo Fisher). Images were acquired at 20x (Olympus 20X/0.7NA) in ‘widefield mode’ and scanned for 25 fields of view per well using laser-based autofocus. The following channels were used: Channel 1, 647 nm (Red) SOX2; Channel 2, 488 nm (Green) NFIA; Channel 3, 647 nm (SOX2 long exposure). SOX2 positive objects were detected and segmented in Channel 1 and used as an estimator of cell number (‘ValidObjectCount’). Channel1 masks were applied to Channel 2 and 3 acquired images and the average intensity per object measured for each channel and summarised as a mean per well (‘MEAN_CircAvgIntenCh2’). The full CRISPR-Cas9 screen was performed twice (biological replicates). For each screen, three technical replicates per gene of interest were used. The processing of raw data was performed using the in-house pipeline based on the celHTS2 pipeline. In brief, non-targeting control (NT) quantifications were used for normalisation across all samples. The average values (intensity, valid object count etc.) of all NT samples were set to 100. The mean normalised intensity of all three technical replicates per gene mutated was calculated. Full downstream analysis can be found at https://github.com/MJDelas/temporal-neural.

### Differentiation timecourse analysis by flow cytometry

Selected hits from the 96-well CRISPR screen were validated in a 12-well secondary screen using flow cytometry. Differentiations were performed in matrigel-coated CellBIND 12-well plates (Corning). CRISPR reaction volumes were calculated relative to the 96-well plate CRISPR mutant screen. 45,000 cells per well were seeded in 500 μl total volume (with a crRNA/tracrRNA complex final concentration of 20 nM). Samples were collected at day 7, 9 and 11 of differentiation. Cells were washed twice with PBS, accutased and transferred from 12-well plates into V-bottom 96-well plates (Corning, 3897). Cells were spun down at 1000 rpm for 4 min and resuspended in PBS plus LIVE/DEAD™ Fixable Dead Cell Stain Near-IR fluorescent reactive dye (Thermo Fisher; L34976) and incubated for 30 min protected from light. Cells were then fixed with 4% PFA for 15 min, washed once with PBS/0.5%BSA and processed for Flow cytometry staining.

### Flow cytometry of intracellular markers

#### Sample collection

Cells were washed three times with PBS before dissociation with Accutase (Gibco, A1110501) for 5 min at 37°C. Cells were collected in ES cell medium, spun down at 400xg for 4 min and resuspended in PBS. Cell suspensions were supplemented with LIVE/DEAD™ Fixable Dead Cell Stain Near-IR fluorescent reactive dye (Thermo Fisher, L34976), according to the manufacturer’s instructions (1 μl dye/1 ml PBS) and incubated for 30 min on ice protected from light. Cells were spun down at 1,000xg for 4 min and fixed with 4% PFA (Thermo Fisher, Cat. No. 28908) for 15 min on ice. After washing once with PBS, cells were resuspended in PBS + 0.5% BSA and stored at 4°C.

#### Staining

Between 1 and 2×10^6^ cells were used for flow cytometry analysis. Cells was transferred into 1.5 ml DNA LoBind® Tubes (Eppendorf) and incubated with 100 μl primary antibodies in blocking solution (PBS + 0.1% Triton X-100 + 1% BSA) overnight at 4°C or if conjugated antibodies were used, samples were incubated for 1-2 hr at RT. The next day, cells were spun down at 4000 rpm for 4 min and washed once with PBS before adding secondary antibodies together with conjugated antibodies for 1-2 hr at room temperature. Cells were washed once with PBST, spun down and resuspended in 300 or 500 μl PBS with 0.5% BSA. Flow cytometry was performed using a Becton Dickinson LSRFortessa™ Cell Analyzer (BD Biosciences) recording 10,000 SOX2-positive cells as stopping gate. Analysis of data was performed using the FlowJo software (Version 10.8.2, Becton Dickinson).

### Immunohistochemistry of embryo sections

Mouse or chicken embryos were fixed in 4% paraformaldehyde for 2 h at room temperature, cryoprotected by equilibration in 0.12 M Sodium Phosphate buffer containing 15%w/v sucrose solution overnight at 4°C. Tissues were subsequently mounted in Sodium Phosphate buffer containing 15% w/v sucrose and 7.5% w/v Gelatin and were flash frozen in Isopentane. Cryosectioning (14 μm sections) was performed on a Cryostat LEICA CM3050S using SuperFrost slides (Thermo Fisher, 10149870).

Immunostaining was performed as previously described,^38^ using the following antibodies. Mouse: mouse anti-NKX6.1 (1:100, DSHB, F55A10), rabbit anti-NFIA (1:1000, Atlas Antibodies, HPA008884), goat anti-OLIG2 (1:500, R&D Systems, AF2418). Chicken: rabbit anti-Olig2 (1:500, EMD Millipore, AB9610), sheep anti-Zfhx3 (1:400, R&D, AF7384), goat-Isl1 (1:500, R&D, AF1837), mouse-Sox2 (1:500, Santa Cruz, sc-365823), rat anti-RFP (1:500, Chromotek, 5f8). Secondary antibodies used were from ThermoFisher conjugated with AlexaFluor and were all used at 1:500 concentration, except AlexaFluor 647 conjugated antibodies that were used 1:1000. Sections were imaged with a Leica SP8 confocal microscope and images processed with Fiji.

### Plasmid generation

To generate the Nr6a1 chicken expression plasmids, we cloned using Golden Gate an IDT gblock containing the ORF of Nr6a1 (Gencode Transcript: ENSMUST00000076275.10), where PaqCI cut sites were removed by synonymous mutation, into an ampicilin resistant backbone, downstream of an Ef1a promoter and upstream of a T2A-mScarlet3 fusion (Addgene 189753), and an SV40 terminator. Our control vector is identical to this but lacking the Nr6a1-T2A, cloned in the same manner. The constructs were transformed using MAX Efficiency™ Stbl2™ Competent Cells (Thermo Fisher 10268019) and were grown in 100mL LB shaking for 16h at 37°C, before maxi-prepping using the QIAGEN Plasmid Plus Maxi Kit (12963).

### Plasmid backbone and virus generation

To generate Nr6a1 lentivirus expression vectors, we amplified by PCR the Ef1a to SV40 inclusive segments of the chicken overexpression plasmids to include Esp3I Golden Gate adaptors using Phusion Flash (Thermo Fisher; F548L), into a lentivirus backbone derived from.^86^ Constructs were transformed using MAX Efficiency™ Stbl2™ Competent Cells (Thermo Fisher10268019) and maxi-prepped as above. To generate lentivirus, HEK293T were plated at 1.5×10^6^ per 6cm plate. The next day, the media was changed (3.5mL). A mixture of 3rd generation packaging plasmids (0.238μg of CMV-Rev, 0.475 μg pMDLg and 0.342 μg VSV-G per 1.9 μl) and 2.28 μg of the transfer plasmid was vortexed with 11.9 μL X-tremeGENE™ HP DNA Transfection Reagent (Roche; 6366244001) and 360 μl OptiMEM (GIBCO; 31985062). This was added dropwise on top of the cells. The media was changed 16h later to N2B27 and collected 30hrs later. The media was filtered through 0.45 micron filter and frozen at -80°C in aliquots.

### RNA extraction and RT-qPCR

Cells were washed twice with PBS and 350 μl RLT lysis buffer (Qiagen, 1015762) was added directly to the dish (e.g. p3.5/35 mm or 1 well of 6-well plate). After 5 min lysed cells were collected and transferred to a 2 ml RNase-free Eppendorf tube and stored at -20°C for no longer than 2 weeks until further processing. Total RNA was extracted using RNeasy Mini Kit with DNase digest (Qiagen, Cat. No. 74106) according to the manufacturer’s instructions and stored at -80°C. 1.5 μg of RNA was reverse transcribed into cDNA using SuperScript III first strand synthesis system (Invitrogen 18080-051) using random hexamers. qRT-PCR was performed in 384-well plates (reaction volume 10 μl) using Platinum™ SYBR™ Green qPCR SuperMix-UDG (Invitrogen A25742) on either the QuantStudio 5 or 12K Flex Real-Time PCR system (ThermoFisher Scientific). Expression values (CT values) were normalised against β-Actin. All experiments were performed in technical duplicates, biological duplicates or triplicates for each time point analysed. Primers were designed using NCBI tool Primer BLAST unless stated otherwise.

### CaTS-ATAC-seq (Crosslinked and TF-Sorted ATAC-seq)

CaTS-ATAC was performed as previously described^38^ with in-house produced Tn5. A dead cell removal step was introduced to enrich for live cells prior to fixation and transposition. The details are as follows: at the differentiation timepoint of collection, cells were washed once with PBS and dissociated with Accutase (Gibco, A1110501) for 5 min at 37°C. Cells were collected in 1.5 ml Lo-Bind Eppendorf tubes (Eppendorf cat. #Z666548) and spun down at 400xg for 4 min. Cell suspensions were supplemented with LIVE/ DEAD™ Fixable Dead Cell Stain Near-IR fluorescent reactive dye (Thermo Fisher, L34976), according to the manufacturer’s instructions (1 μl dye/1 ml PBS for 1×10^6^ cells) and incubated for 30 min on ice protected from light. During the incubation, the autoMACS® Pro Separator (Miltenyi Biotec) was set-up according to the manufacturer’s instructions. After the 30 min cell suspensions were divided into 15 ml falcon tubes and spun down for 10 min at 400xg at 4°C. Cells were resuspended, pooled into one tube, and counted. Cells were magnetically labelled with Dead Cell Removal Microbeads from the Dead Cell Removal Kit (Miltenyi Biotec, 130-090-101). For 1.0×10^7^ cells, 100 μl beads were used. Cells and microbeads were mixed and incubated for 15 min at room temperature. If necessary, 1x Binding Buffer provided by the kit was added to the cell suspension to reach a minimum volume of 500 μl for separation. The samples were run through the autoMACS® Pro Separator (Miltenyi Biotec) using the ‘Depl05’ programme. The microbeads label dead cells, which are retained in the separation columns. Unlabelled live cells run through the column and were collected in a 15 ml falcon tube in PBS. Live cells were count and spun down for 4 min at 400xg at 4°C. Cell pellet was resuspended in 300 μl of PBS in 1.5 ml LoBind Eppendorf tubes. Cells were then fixed, transposed, stained for viability and intracellular markers (anti-SOX2-V450, anti-Nkx6.1-PE, goat anti-Olig2, rabbit anti-Nfia) and sorted. DNA of samples was isolated using the DNA Clean & Concentrator-5 kit (Zymo; D4013) as per manufacturer’s instructions.

### CaTS-RNA-seq (Crosslinked and TF-Sorted RNA-seq)

Samples for CaTS-RNA-seq were collected in the same way as for CaTS-ATAC-seq until the fixation step. From there on all buffers were supplemented with 1:100 RNasin Plus RNase inhibitor (Promega, Cat. No. N2615).

#### Glyoxal fixation

The fixation protocol was adapted from published work.^87^ To prepare ~4 ml of 3% glyoxal solution, 2.835 ml of RNase-free water was combined with 0.789 ml 100% (v/v) EtOH, 0.03 ml acetic acid and 0.313 ml 40% Glyoxal (Sigma-Aldrich, Cat. No. 50649-100ML). All steps were performed on ice and reagents were cooled before mixing. The solution was adjusted to pH 4-5 (with 1M NaOH) if necessary. The solution was always kept on ice and stored for up to 3 days at 4°C. All samples were fixed and stained on the same day. To minimise RNase activity, cells were kept on ice and only pre-chilled reagents were used.

Cells were resuspended in 50 μl PBS + 0.5% BSA first, followed by adding 300 μl 3% glyoxal. Cells were fixed for 15 min with slow side-to-side rocking on ice protected from light. 25 μl of 2M glycine was added for quenching and incubated for 5 min. 1 ml PBS + 0.5 % BSA was added to stop the fixation, and cells were spun down at 2000xg for 5 min at 4°C. 1-2×10^6^ cells were prepared for Flow cytometry staining and resuspended in 100 μl PBS + 0.5% BSA + 0.3% Triton X-100. Cells were incubated on ice for 30 min. Staining was performed as previously described and processed in the same way as described for CaTS-ATAC-seq. 10-15,000 cells of each desired population were sorted into 300 μl TRIzol reagent (Invitrogen, Cat. No. 15596026) and stored at -80°C until further processing.

#### TRIzol RNA extraction and library preparation

All steps were performed at room temperature unless otherwise stated. For TRIzol extraction, Phasemaker tubes (Thermo Fisher A33248) were used according to the manufacturer’s instructions. 300 μl of sorted cells in TRIzol were mixed and transferred to Phasemaker tubes and incubated for 5 min at room temperature. 200 μl chloroform per 1 ml TRIzol used for lysis was added and mixed vigorously. Samples were incubated for 10 min and spun for 5 min at 12,000×g and 4°C. The upper phase was transferred to a new RNase-free tube. 8 μg RNase-free GlycoBlue (stock 15 mg/ml) was added to each sample and briefly mixed to see the pellet easier. 150 μl of 100% isopropanol was added (500 μl per initial 1 ml TRIzol). The solution was incubated for 10 min, then centrifuged for 10 min at 12,000xg at 4°C and supernatant was carefully discarded. The pellet was washed in 1 ml of 75% EtOH per 1 ml TRIzol reagent used for lysis. The sample was then centrifuged for 5 min at 7500xg at 4°C. The pellet was air dried for 5-10 min to remove residual EtOH. To solubilise the pellet, it was resuspended in ~17 μl of RNase-free water. The resuspended RNA was incubated in a heat block for 10 min at 55°C. RNA was stored at -80°C until further processing.

After analysis RNA integrity using the Agilent Bioanalyser, paired-end library preparation was performed using the SMART-Seq HT kit (Takara, Cat. No. 634437) followed by Nextera XT DNA Library Preparation Kit (Illumina, Cat. No. FC-131-1096). Indexed libraries were pooled and sequenced on an Illumina HiSeq 4000 flow cell configured to generate 100 bp of pair-ended (PE) data (Number of reads per sample: 25 million pairs).

### Bulk RNA-seq from CRISPR-transfected mutants

Samples were collected at day 7, 9 and 11 of differentiation and transferred from 12-well plates into 96-well plates (see above). Cells were spun down and pellets stored at -80°C. Total RNA was extracted using RNeasy Mini Kit with DNAse digest (Qiagen, Cat. No. 74106) according to the manufacturer’s instructions and stored at -80°C. Libraries were prepared using the NEBNext Ultra II Direc-tional kit(NEB, Cat no E7760L) with ribodepletion (Qiagen QIAseq FastSelect -rRNA HMR Kit, Cat. No. 334386), following manufacturer’s instructions.

### Bulk ATAC-seq from lentiviral overexpressing cells

Differentiations were transduced at day 5 with lentivirus overexpressing either *Ef1a:mScarlet3* or *Ef1a:Nr6a1-T2A-mScarlet3*. Three independent differentiation wells were transduced as biological replicates. At day 9, live mScarlet3+ positive cells were sorted and 50,000 cells per replicate were used for ATAC-seq library preparation. Bulk ATAC-seq on live cells was performed as previously described.^38^

### Chicken embryos and electroporation

Fertilized chicken embryos (*Gallus gallus*) were obtained from Henry Stewart & Co. Ltd and kept in humidified incubators at 38°C up to the desired Hamburger and Hamilton (HH) stage. Eggs were incubated for 40-48 h to reach HH11-HH12 stages and injected in the developing neural tube via a pulled glass capillary (Harvard Apparatus, EC1 64-0766) with plasmid solutions mixed with SYBR Green (Thermo Fisher, S7563). *Ef1a:mScarlet3* or *Ef1a:Nr6a1-T2A-mScarlet3* plasmids were injected at a final concentration of 150-300 ng/ μl, according to the concentration that ensured consistent mScarlet3 expression. Electroporations were performed as previously described^88^ by passing three 24 V pulses for 50 ms each every 150 ms with an Electro Square Porator ECM830. The embryos were then incubated until stage HH23 (day 4) or HH25 (day 5) and screened for transfection efficiency (i.e., presence of mScarlet3 fluorescence within the neural tube). Healthy and correctly transfected embryos were dissected, by selecting brachial regions of the trunk, and processed for immunohistochemistry.

### Quantification And Statistical Analysis

#### ATAC-seq processing

Data was processed using the nf-core atacseq pipeline (https://nf-co.re/atacseq) with the following options: –genome mm10 –skip_ diff_analysis –min_reps_consensus 2 -r 1.2.1.

#### RNA-seq processing

Data was processed using the nf-core rnaseq pipeline with the following options: –genome mm10 –aligner star_salmon –skip_biotype_qc –deseq2_vst -r 3.5.

#### ATAC-seq differential expression analysis

Read counts within the consensus intervals generated by featureCounts were used as input for DESeq2.^84^ Principal Component Analysis was performed using the top 30000 most variable elements and coloured by different sample metadata.

To assess the cell type specific accessibility for the same NP cell types at different timepoints, or the differential accessibility between timepoints for the same cell type, pairwise differential expression was performed. The number of differentially accessible intervals plotted (fold change > 2, basemean > 100, p-adj < 0.01) as a bubble plot.

To assemble the subset of highly-confident global temporally dynamic elements, differentially accessible (thresholds as above) elements in all three cell types (but in any number of temporal comparisons) were selected.

Elements affected by Nfia/Nfib double knockout were identified using the same thresholds in KO vs wildtype comparisons.

All significantly differentially accessible elements (no fold change cut off) between control and Nr6a1 overexpression are plotted coloured by p-adjusted value.

#### ATAC element clusters by kmeans

Variance stabilized transformed data generated using DESeq2 were used as input to identify clusters of elements with the same dynamics. Clustering was performed using kmeans with a high number of centres, 30, and subsequently re-grouping clusters of very similar dynamics using hclust and target of 7 final clusters. This was chosen as independent iterations resulted in reproducible clusters and dynamics.

#### Footprinting analysis

BAM files for merged replicates were used as input for TOBIAS ATACorrect,^60^ followed by TOBIAS Footprint. TOBIAS BINDetect was run on all samples combined using JASPAR2018, HOCOMOCO and Taipale databases.^89–91^ This was performed using and Nextflow TOBIAS wrapper https://github.com/luslab/briscoe-nf-tobias. Motifs that were amongst the top 5% highest absolute fold change or 5% smallest *p*values in each pair-wise comparison were selected. Motifs were grouped into archetypes.^61^

#### Motif enrichment analysis

Elements differentially accessible between day 7 and day 11 were split into NFIA/NFIB-dependent or independent if they were differentially accessible at day 11 in the dKO cells or not, respectively. The AME tool^92^ was used as part of the R interface for the MEME suite (https://github.com/snystrom/memes). The same motif databases as those used for footprinting were used as input. Motifs were grouped into archetypes to avoid reporting of the same motif.

#### RNA-seq differential expression analysis

The read counts from salmon were used as input for DESeq2. The threshholds used to consider a gene differentially expressed were filter(padj < 0.05 & abs(log2FoldChange) > 1 & baseMean > 80). To assemble the subset of global dynamic genes, we selected those differentially expressed in all cell types but in any number of temporal comparisons. To assemble the list of genes temporally dynamic but also cell type specific, we required genes to be differentially expressed either (i) at more than one timepoint and in at least one cell type comparison or (ii) in at least one timepoint and in two cell type comparisons.

The effects of the different mutations were examined by performing differential expression between control and KO at each timepoint.

#### Analysis of temporal gene clusters

Global temporally dynamic genes were clustered using ComplexHeatmap^93^ into 6 reproducible clusters (Yu et al., 2012).

#### Association of genes with candidate elements

The genes identified as temporally dynamic and also differentially expressed between cell types were used as input for figR’s function runGenePeakcorr with windowPadSize = 500000.^94^ The gene to peak correlation assignments were performed independently for each cell type. For each element-gene pair, the most significant, or most correlated in case of equal pval, cell type values were kept. The accessibility of elements assigned to input genes with pvalz < 0.1, rObs > 0 was plotted. For visualization purposes, genes were clustered and their assigned genes plotted in the same order.

#### Reanalysis of scATACseq across the neuraxis

In vivo spinal cord single cell data^56^ were downloaded as fastq and processed using CellRanger-ATAC/2.1.0 cellranger-atac count. downloaded as processed bigwig files and the coverage for the spinal cord temporal elements was plotted using deeptools function. Data from other regions^3,11,57,58^ were downloaded as mapped files. Fragment files from^56^ were imported into ArchR.^85^ Batch correction was performed using Harmony (addHarmony) and clusters were defined using addClusters (method = “Seurat”, resolution = 0.8). Clusters were identified as neural progenitors based on high gene score for Sox2, and low score for markers of neurons (Tubb3 and Elav3) or neural crest (Sox10). For the rest of the datasets, the cells identified as progenitors in the authors’ deposited metadata were used to filter the cells from the appropriate clusters. Normalized bigwig files for neural progenitors at each developmental stage were exported from ArchR. Deeptools plotHeatmap^95^ were used for data visualization. scATAC values for the in vitro-defined temporal program were extracted for the progenitors of each timepoint and dataset using ArchR getGroupSE(), and Pearson’s correlations and *p*-values were calculated using the Hmisc’s rcorr() function. Code available in github repository.

## Supplementary Material

Table S1

Table S2

Table S3

Table S4

Table S5

Table S6

Supplementary Material

## Data Availability

Sequencing data (CaTS-ATAC, CaTS-RNA-seq, and bulk ATAC-seq) have been deposited at GEO and are publicly available as of the date of publication. Accession numbers are listed in the key resources table. Accession number for the SuperSeries is GEO: GSE264172. Microscopy data reported in this paper will be shared by the lead contact upon request. The code is available at https://github.com/MJDelas/temporal-neural. Any additional information required to reanalyze the data reported in this work paper is available from the lead contact upon request. All the sequencing data asscociated with this study is available at GEO SuperSeries GEO: GSE264172.

## Highlights

- A temporal program of chromatin accessibility operates across CNS progenitors
- This program integrates spatial inputs to direct cell-type-specific gene expression
- A CRISPR screen identifies Nr6a1 and other regulators of the temporal program
- Disrupting the temporal chromatin program affects neuronal subtype generation

## In brief

Zhang et al. identify a global temporal chromatin program that operates across the developing vertebrate nervous system to control neural cell diversity. This mechanism, which involves Nr6a1 and NFIA/B, integrates spatial and temporal patterning through sequential chromatin accessibility changes that direct when and where specific cell types are generated.
