# Supplementary material for: The cis-regulatory logic integrating spatial and temporal patterning in the vertebrate neural tube
Source: Dev Cell. Author manuscript; Available in PMC 2026 Jul 14. (PMC7619239; doi:10.1016/j.devcel.2025.06.029)
Supplement: Supplementary Material [file EMS214911-supplement-Supplementary_Material.pdf]

Developmental Cell, Volume *60*

Supplemental information

The *cis*-regulatory logic integrating spatial and temporal patterning in the vertebrate neural tube

Isabel Zhang, Giulia L.M. Boezio, Jake Cornwall-Scoones, Thomas Frith, Elizabeth Finnie, Junyi Luo, Ming Jiang, Michael Howell, Robin Lovell-Badge, Andreas Sagner, James Briscoe, and M. Joaquina Delás

Supplemental figures

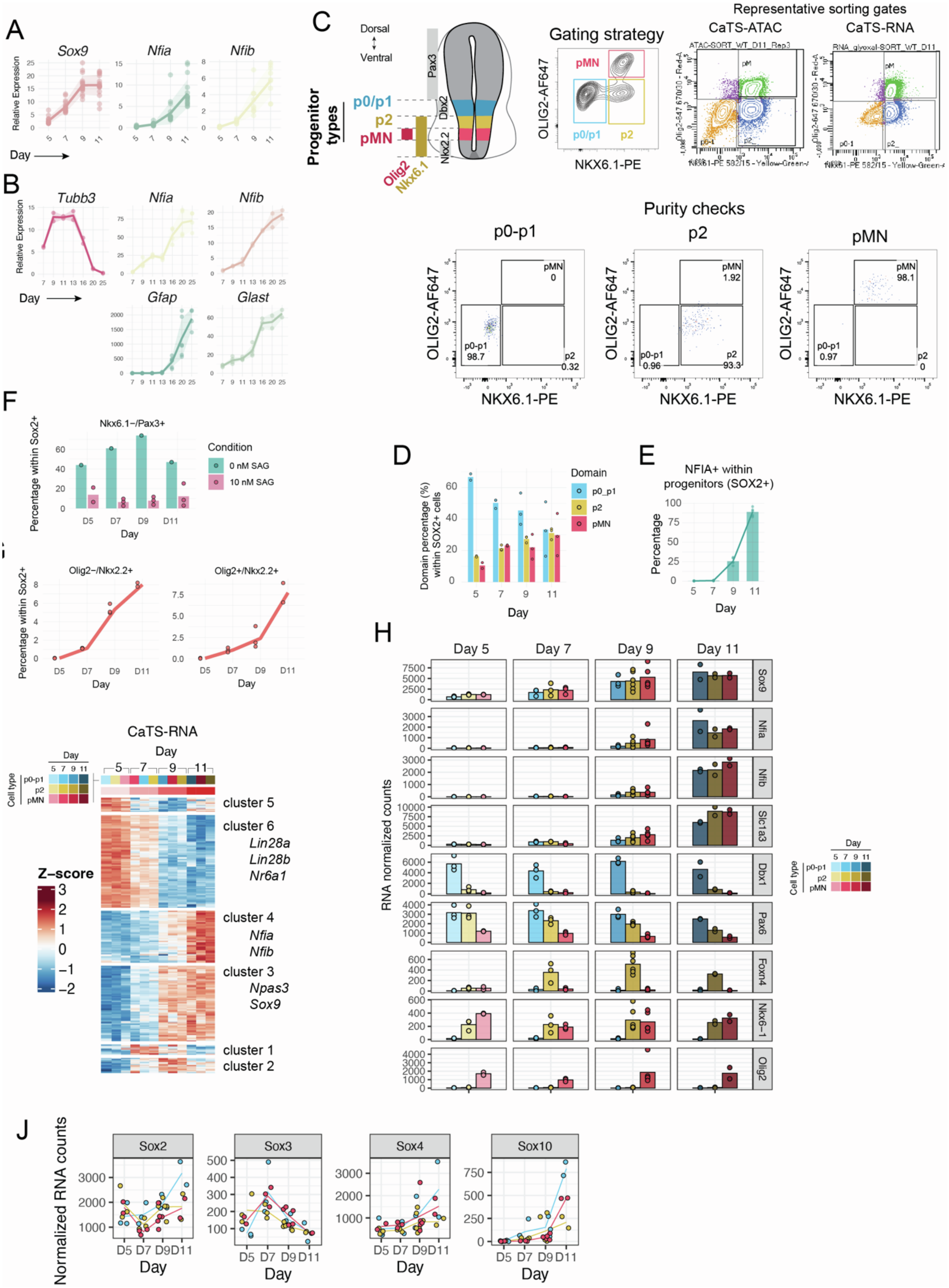

Supplemental Figure 1: Mouse ES cell differentiations recapitulate temporal progression features. Related to Figure 1.

(A) RT-qPCR highlighting key genes in the temporal progression to day 11, and
(B) to day 25. Each dot is an individual sample, line is the average and shading depicts the mean +/- standard deviation for each gene at each timepoint.
(C) Diagram of the spatial domains analysed in this study and the markers for the cell types of interest and the neighbouring domains (left), and example flow cytometry plot for the cell types of interest ("Sorting strategy"). Representative sorting gating for p0-p1, p2 and pMN for both CaTS-ATAC and CaTS-RNA are shown (right). Representative purity checked shown below.
(D) Proportions of cell types (spatial domains) obtained in the cellular model over the time course progression, as measured by flow cytometry. Dots are individual measurements from independent differentiations, bars reflect the mean value.
(E) Proportion of NFIA in the cellular model as measure by flow cytometry. Dots are individual measurements from independent differentiations, bars and line reflect the mean values.
(F) Proportion of cells dorsal to p0 (PAX3+) was very low in the conditions used for this study (10 nM SAG). Proportions obtained with 0 nM SAG are shown for comparison. Dots are individual measurements from independent differentiations, bars reflect the mean value.
(G) Proportion of cells ventral to pMN (NKX2.2+) were only detected at very low levels at the later timepoints (left). NKX2.2 is known to be induced as part of oligodendrocyte progenitor specification and some co-expression of Olig2 and Nkx2.2 is present as expected. Dots are individual measurements from independent differentiations, lines reflect the mean value.
(H) Expression of marker genes in the CaTS-RNA dataset, for each cell type and timepoint as further validation of the cellular model. Dots are individual measurements from independent differentiations, bars reflect the mean value.
(I) Global gene expression over time shows differential gene expression at different timepoints.
(J) Normalised RNA expression of additional genes that could contribute to the SOX/1 footprint. Dots are individual measurements from independent differentiations, lines reflect the mean value.

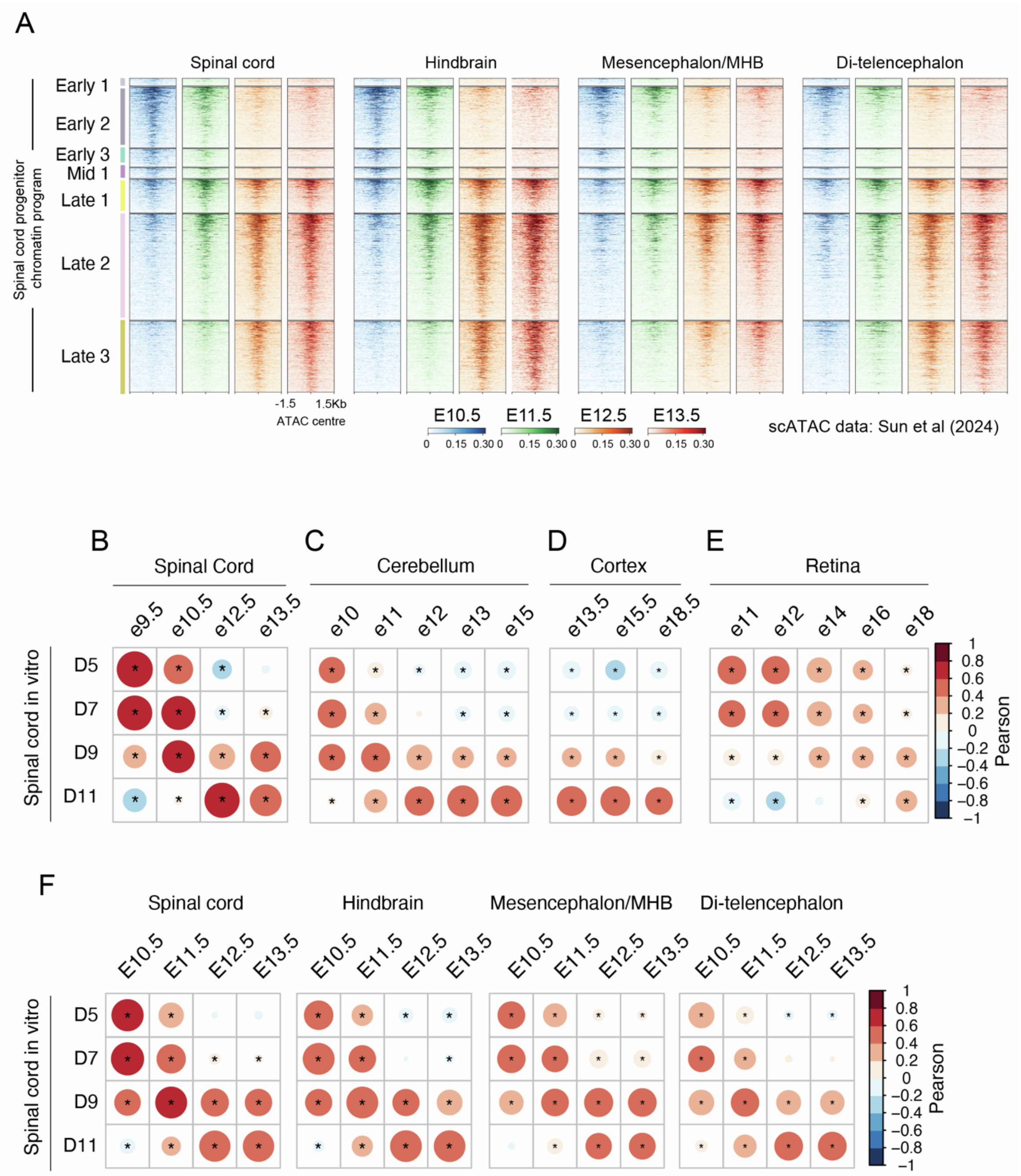

Supplemental Figure 2: The global chromatin temporal program across CNS regions. Related to Figure 2.

(A) Heatmap coverage plots of accessibility at the temporal chromatin elements for cells from the spinal cord, hindbrain, mesencephalon and di-telencephalon. The elements plotted are those from the spinal cord progenitor temporal programme (Fig 2A; Shu et al., 2022). Data are from (Sun et al., 2024)

(B-E) Pearson correlation coefficient between each in vitro timepoint (rows) and the in vivo timepoints for progenitors across the developing spinal cord (Fig 2B), cerebellum, cortex and retina (Fig 2C-D) shows high concordance of expected samples.

(F) Pearson correlation coefficient between in vitro timepoints and in vivo progenitors from the organogenesis atlas (Fig S2A) shows high concordance across CNS regions. * denotes pvalue < 0.01 approximated using the t distribution.

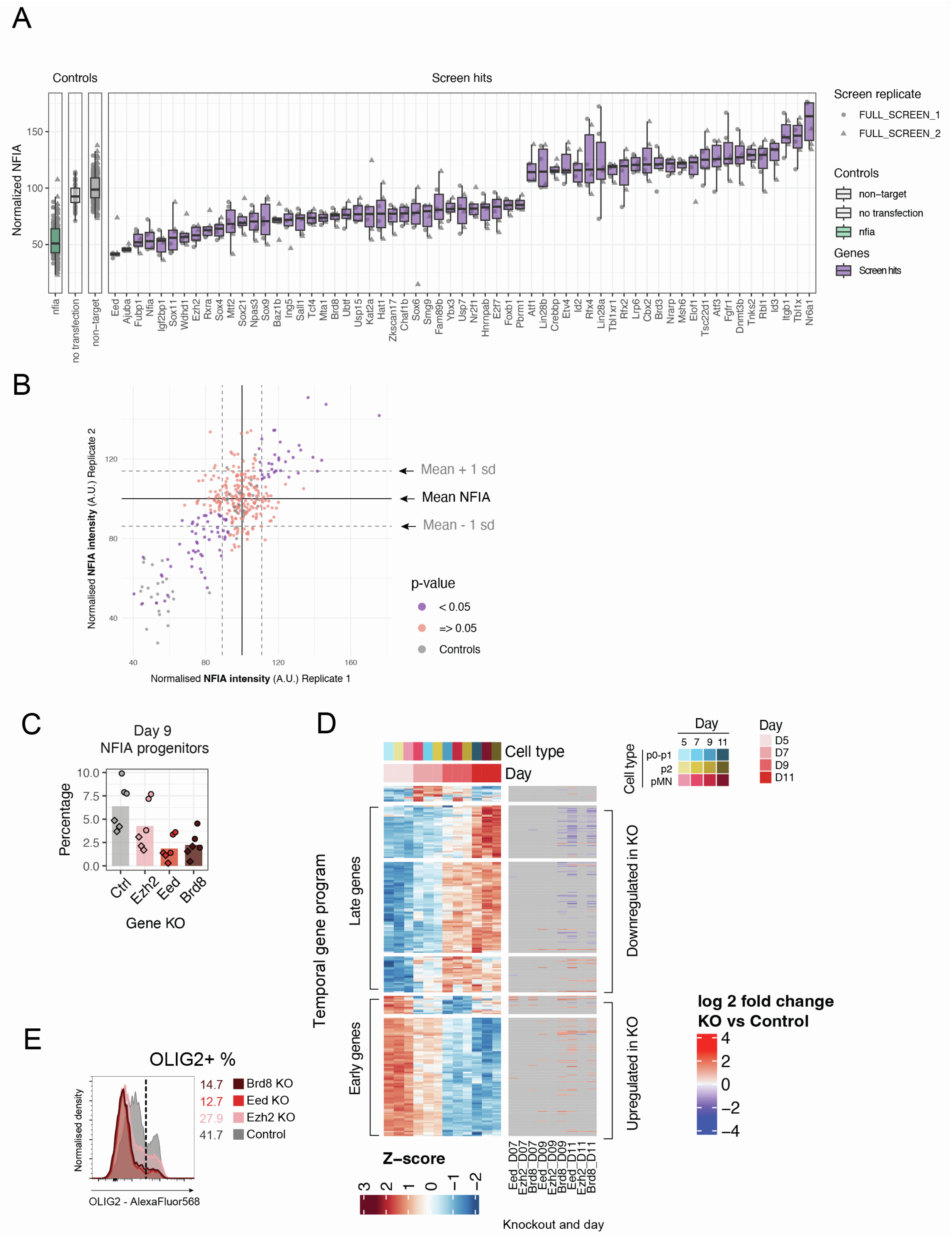

Supplemental Figure 3: CRISPR screen results and characterization of key candidates. Related to Figure 3.

(A) Normalised NFIA intensity for all technical replicates in each of the two biological replicates of the CRISPR screen for all controls and genes selected as hits. Boxplots correspond to the median and the 25 th and 75 th percentiles. The whiskers extend to the largest values, but no further than 1.5, the interquartile range (distance between the first and third quartiles), in

which case the outliers are shown. Circles or triangles show each individual measurements for screen repeat 1 and 2, respectively.

(B) Screen results showing normalized NFIA intensity across both replicates (same as Fig 3D) coloured by p-value < 0.05, over, or control (Wilcoxon–Mann–Whitney test). Solid lines represent the mean value of the negative control in each replicate (normalized to 100), dashed lines show the mean +/- 1 standard deviation for each replicate.

(C) Flow cytometry staining showing the proportion of NFIA + cells within the SOX2 NPs for each of the indicated knockouts at day 9. Dots are individual measurements from independent differentiations, bars reflect the mean value.

(D) Knockout of either Eed, Ezh2 or Brd8 results in changes in genes identified as part of the temporal program, with late genes being downregulated and early genes being upregulated in knockout compared to control. The left heatmap is the same data and clustering as Fig S1I, the temporal genes with their normalized relative expression across time and cell types. On the right, we show the fold change in mutant vs control for each day and perturbed gene (Eed, Ezh2, Brd8) for the significant changes (red if gene is upregulated, blue if gene is downregulated). A grey box means the gene is not significantly differentially expressed in that timepoint of mutant.

(E) The proportion of OLIG2+ progenitor cells is reduced when *Ezh2, Eed* or *Brd8* are knocked out compared to non-targeting controls. Data shown for day 11.

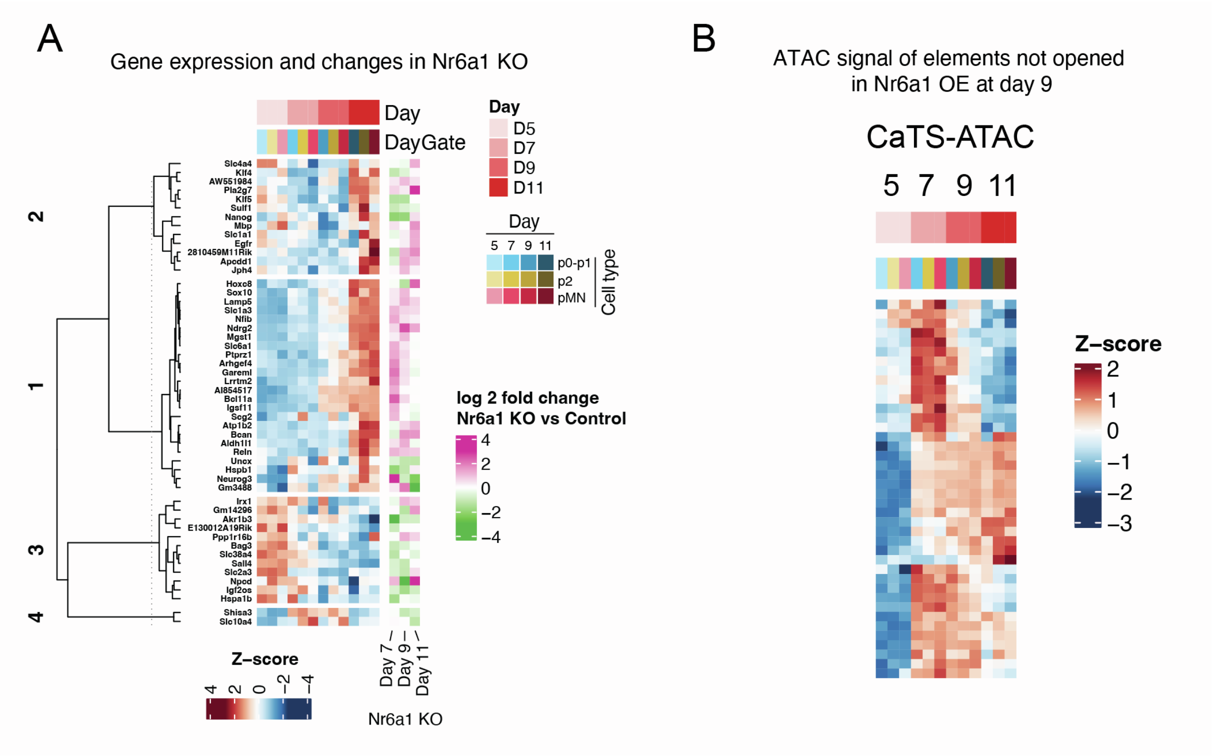

Supplemental Figure 4: Nra6a1 promotes the early program. Related to Figure 4.

(A) Heatmap of genes affected by Nr6a1 KO showing the expression during normal differentiation (left) and the changes observed in Nr6a1 KO compared to control (right).

(B) Accessibility z-scores of the elements during the normal differentiation identified as only opened in control but not in Nr6a1 overexpression.

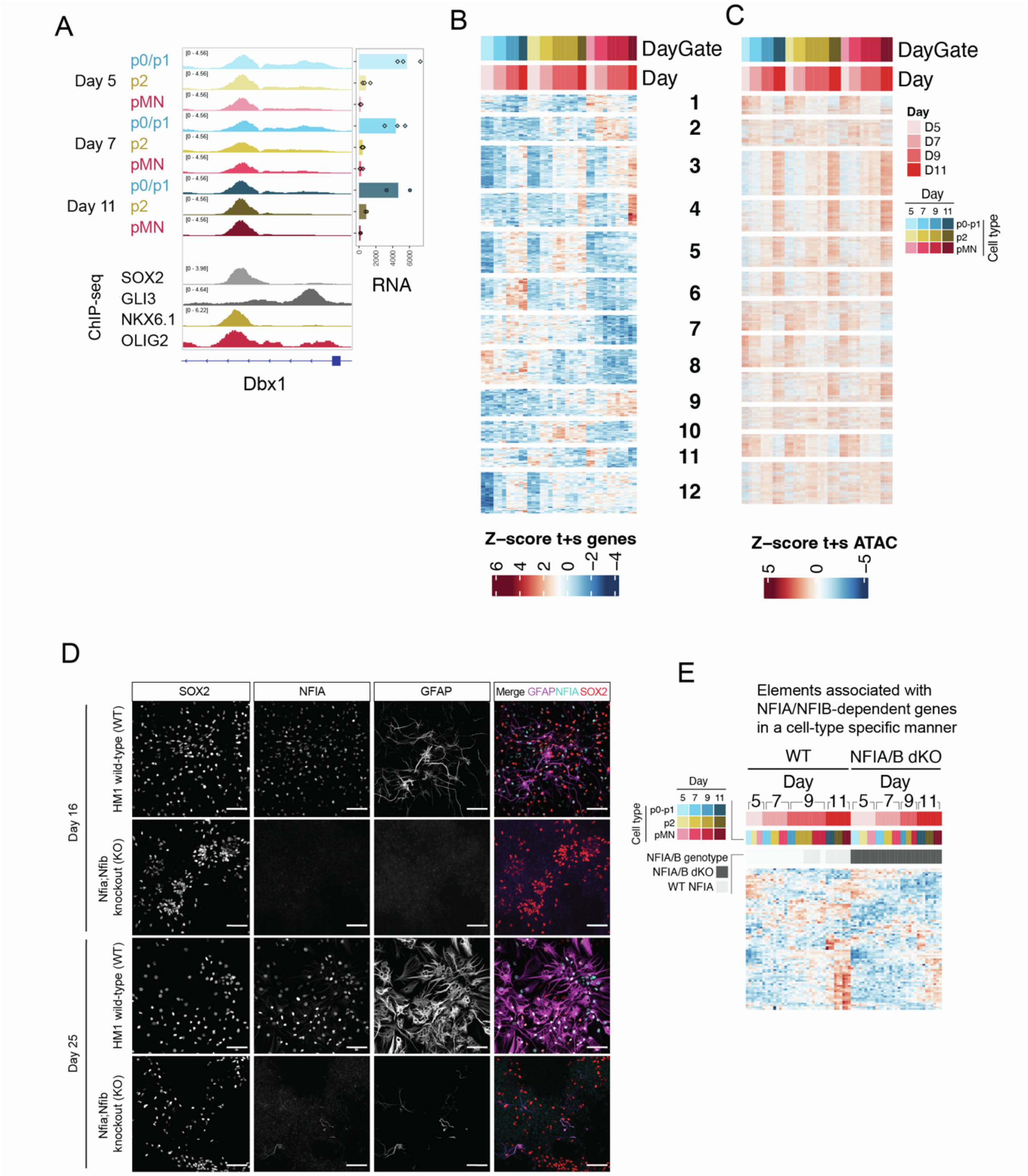

Supplemental Figure 5: The global temporal program controls cell type-specific genes. Related to Figure 5.

(A) ATAC-seq accessibility and RNA expression of Dbx1 in the different cell types and timepoints shows global accessibility across cell types despite the cell type specific gene expression. ChIP-seq shows the element is bound by cell type specific repressors, global activators and effects of the Shh pathway. For RNA, dots are individual measurements from independent differentiations, bars reflect the mean value.

(B) Genes identified as being dynamically regulated over time and differentially expressed between cell types, clustered into 10 groups of different expression dynamics.

(C) Predicted regulatory elements associated with the genes in (B) show dynamic temporal accessibility but always consistent across all cell types.

(D) The ability of Nfia/Nfib dKO to upregulate the astrocytic marker GFAP is greatly impared during in vitro differentiation. Scale bar 60 μm.

(E) Elements associated with NFIA/NFIB-dependent genes affected in a cell type-specific manner loose accessibility in the NFIA/NFIB dKO cells.
